# Perceptions about children and adolescents’ mental health crisis intervention: a qualitative systematic review

**DOI:** 10.1590/0102-311XEN016324

**Published:** 2024-12-20

**Authors:** Nathalia Nakano Telles, Nathalia dos Santos Cruz, Marilia Mastrocolla de Almeida Cardoso, Priscilla de Oliveira Luz, Heloísa Garcia Claro Fernandes, Márcia Aparecida Ferreira de Oliveira

**Affiliations:** 1 Escola de Enfermagem, Universidade de São Paulo, São Paulo, Brasil.; 2 Hospital das Clínicas da Faculdade de Medicina de Botucatu, Universidade Estadual Paulista Júlio de Mesquita Filho, Botucatu, Brasil.; 3 Universidade Estadual de Campinas, Campinas, Brasil.

**Keywords:** Mental Health, Crisis Intervention, Child, Adolescent, Perception, Saúde Mental, Intervenção em Crise, Criança, Adolescente, Percepção, Salud Mental, Intervención en la Crisis, Niño, Adolescente, Percepción

## Abstract

This review aimed to identify and synthesize the perceptions of mental healthcare professionals, family members, and users about mental health crisis interventions for children and adolescents at hospitals and community mental health services. A qualitative systematic review was conducted following the Joanna Briggs Institution guidelines. The search was performed in 15 databases, with no temporal delimitation, and included studies in Portuguese, English, and Spanish. All works were assessed regarding methodological quality, credibility, and dependability according to the ConQual score and the recommendations were assessed following the Joanna Briggs Institution guidelines. In total, two independent reviewers screened and assessed the studies, extracted their data, developed categories, and conducted the thematic synthesis. A total of 13 studies met the inclusion and exclusion criteria. From these, five syntheses were developed: importance of relationships; importance of procedures during treatment; positive emotional responses to treatment; negative emotional responses to treatment; and issues with health professionals and health services. All five syntheses presented high dependability; two syntheses presented high credibility; and three presented moderate credibility. Mental healthcare professionals, family members and users had convergent perceptions about crisis intervention provided at healthcare services. Understanding their perceptions to improve care and the user experience in this vulnerable situation is crucial.

## Resumo

Esta revisão teve como objetivo identificar e sintetizar as percepções de profissionais de saúde mental, familiares e usuários sobre a intervenção em crises de saúde mental de crianças e adolescentes em hospitais e serviços comunitários especializados. Uma revisão sistemática qualitativa foi conduzida de acordo com as diretrizes do Instituto Joanna Briggs. A busca foi feita em 15 bases de dados, sem delimitação temporal, selecionando estudos nos idiomas português, inglês e espanhol. Todos os estudos incluídos foram avaliados quanto à qualidade metodológica, credibilidade e confiabilidade de acordo com o escore ConQual e as recomendações foram avaliadas pelas diretrizes do Instituto Joanna Briggs. Dois revisores independentes examinaram e avaliaram os estudos, extraíram dados, e elaboraram categorias e síntese temática. Ao todo, 13 estudos atenderam aos critérios de inclusão e exclusão. Foram elaboradas cinco sínteses: importância dos relacionamentos; importância dos procedimentos durante o tratamento; respostas emocionais positivas ao tratamento; respostas emocionais negativas ao tratamento; e questões sobre a equipe de saúde e os serviços de saúde. Todas as cinco sínteses apresentaram alta confiabilidade; duas sínteses apresentaram alta credibilidade; e três apresentaram credibilidade moderada. Profissionais de saúde mental, familiares e usuários apresentaram percepções convergentes sobre a intervenção em crises prestada nos serviços de saúde. É fundamental entender as percepções do usuário para melhorar seu atendimento e experiência nessa situação vulnerável.

## Resumen

Esta revisión tuvo como objetivo identificar y sintetizar las percepciones de profesionales de salud mental, familiares y usuarios sobre la intervención en crisis de salud mental de niños y adolescentes en hospitales y servicios comunitarios especializados. Se realizó una revisión sistemática cualitativa de acuerdo con las directrices del Instituto Joanna Briggs. La búsqueda se realizó en 15 bases de datos, sin delimitación temporal, seleccionando estudios en portugués, inglés y español. Todos los estudios incluidos se evaluaron en cuanto a la calidad metodológica, la credibilidad y la confiabilidad de acuerdo con la puntuación ConQual y las recomendaciones se evaluaron utilizando las directrices del Instituto Joanna Briggs. Dos revisores independientes examinaron y evaluaron los estudios, extrajeron datos y elaboraron categorías y síntesis temáticas. En total, 13 estudios cumplieron con los criterios de inclusión y exclusión. Se elaboraron cinco síntesis: importancia de las relaciones; importancia de los procedimientos durante el tratamiento; respuestas emocionales positivas al tratamiento; respuestas emocionales negativas al tratamiento; y preguntas sobre el equipo de salud y los servicios de salud. Todas las cinco síntesis mostraron una alta confiabilidad; dos síntesis presentaron alta credibilidad; y tres presentaron una credibilidad moderada. Profesionales de salud mental, familiares y usuarios presentaron percepciones convergentes sobre la intervención en crisis brindada en los servicios de salud. Es fundamental comprender las percepciones de los usuarios para mejorar su atención y experiencia en esta situación vulnerable.

## Introduction

Data from the World Health Organization (WHO) reveal that approximately 8% of children aged 5 to 9 years and 14% of adolescents have a mental health problem that persists into adulthood in 50% of cases if inadequately treated ^1^. This represents around 86 million adolescents aged 15 to 19 and 80 million aged 10 to 14 ^2^. Despite this, investments intended to public policies for child and adolescent mental health care are still incipient worldwide ^3^.

Care in this field is complex and, thus, must be carried out in conjunction with various knowledge. This leads the care team to be composed of professionals from multiple areas to favor the aggregation of different perspectives ^4^ ^,^ ^5^. In this sense, it is important that these professionals are heard and that their perceptions concerning daily practices are considered to achieve a better quality of care ^6^.

Family members are essential in the support network for people with mental health problems, especially in the community care model. It is crucial to consider their experiences, demands, and needs ^7^. Moreover, valuing the perspective of children and adolescents is crucial, as their experiences indicate their understanding of health and the quality of care received ^8^.

People with mental health problems may face crises, which are moments of vulnerability ^9^. To better understand these crises, studies focus on the perceptions of those involved: professionals, family members, and users ^10^ ^,^ ^11^ ^,^ ^12^ ^,^ ^13^ ^,^ ^14^ ^,^ ^15^. Generally, mental health crises are treated in long-stays hospitals for a punctual moment or via community services, in which longitudinal care articulated to the user’s environment is achievable ^1^ ^,^ ^16^. Understanding these two methods of care and listening to those affected is essential.

We highlight that a preliminary search was conducted in PROSPERO (International Prospective Register of Systematic Reviews), MEDLINE, Cochrane Database of Systematic Reviews, and JBI Evidence Synthesis databases and no records of systematic reviews like this were found. This review aimed to identify and synthesize the perceptions of mental health professionals, family members, and users regarding the interventions carried out in crisis situations involving children and adolescents at hospitals and community mental health services.

## Method

### Design

This qualitative systematic review was conducted following the Joanna Briggs Institute (JBI) methodological guidelines ^17^. The systematic review design was chosen since it enabled reaching a question of relevance that should be asked and answered via primary studies by identifying and synthesizing these findings ^18^.

This review was registered on the PROSPERO (CRD42022374822) and the review protocol was published elsewhere ^19^.

### Review question, eligibility criteria, and search strategy

The review question was developed following the PICo strategy, in which P refers to population: mental healthcare professionals, family members, and users; I refers to interest phenomena: perceptions about children and adolescents’ mental health crisis intervention; and C refers to the context: hospitals and community mental health services. This led to the question: “what are the perceptions of mental healthcare professionals, family members, and users about children and adolescents’ mental health crisis intervention at hospitals and community mental health services?”.

Eligibility criteria included primary qualitative studies, fully available in Portuguese, English, or Spanish, in which the target care population was children and/or adolescents, considering adolescents as people up to 19 years old ^20^. Studies that presented adults in the sample were excluded, as well as studies exclusively about children and adolescents who declared consuming alcohol and/or other drugs. No temporal delimitation or specific study designs were determined.

Via the keywords that make up the review question, the descriptors used in the Medical Subject Headings (MeSH), Emtree, and Health Sciences Descriptors (DeCS, acronym in Portuguese) were selected. The Boolean operators OR and AND were used to combine descriptors in each database and the “advanced search” tool was used in the databases. The Supplementary Material (Box S1; https://cadernos.ensp.fiocruz.br/static//arquivo/suppl-e00016324_6066.pdf) shows the search strategy employed in each database.

### Study search and selection

The search was conducted by two independent reviewers in December 2022 in 15 databases: Embase, Scopus, Web of Science, Cummulative Index to Nursing & Allied Health Literature (CINAHL) via EBSCO, PubMed, Virtual Health Library (VHL), PsycInfo, and Cochrane Central Register of Controlled Trials (CENTRAL). To identify grey literature, a search was conducted in the portals Brazilian Digital Library of Theses and Dissertations (BDTD, acronym in Portuguese), CAPES Thesis and Dissertations Database, DART-Europe E-theses Portal (DART-E), Cybertesis, Google Scholar, Open Access Theses and Dissertation (OATD), Database of African Theses and Dissertations, and ProQuest. Additionally, citation search was conducted.

The retrieved studies were imported to EndNote (http://www.endnote.com/) to remove duplicate studies and, in the next step, studies underwent initial selection by two independent reviewers using the Rayyan software (https://www.rayyan.ai/) ^21^. Selection conflicts were solved by consulting a third reviewer. After fully reading the selected studies, the process was documented in the PRISMA (*Preferred Reporting Items for Systematic Reviews and Meta-Analyses*) 2020 flowchart ^22^.

### Methodological quality of the studies and data analysis

The final sample was characterized and methodologically assessed following the JBI guidelines ^23^ ^,^ ^24^.

In the following step, findings and illustrations were selected and grouped into categories following the JBI meta-aggregation method ^17^, classifying them as “unequivocal”, “credible”, or “not supported” based on the JBI Credibility Levels ^25^. Only findings classified as “unequivocal” or “credible” were included in the final review. This process resulted in a comprehensive set of findings presented in the form of a descriptive synthesis.

The studies were assessed independently by two reviewers using the *JBI Critical Appraisal Skills Program Qualitative Research Checklist* ^17^. This process was conducted in the SUMARI software (https://sumari.jbi.global/) ^24^, and all studies were included regardless of the answers to each question on the instrument. The result of this step was used to support the ConQual framework in the summary of findings, the discussion stage, and the limitations of this review. To assess the confidence of the synthesized qualitative findings, they were classified following the ConQual approach ^25^, classifying the studies as high, moderate, low, and very low. Dependability is based on the first five questions of the instrument related to research adequacy. The classification varies on the “yes” responses: (i) 4 to 5, the paper remains unchanged; (ii) 2 to 3, moves down one level; and (iii) 0 to 1, moves down two levels. The credibility is scored as unequivocal (U), credible (C), or not supported (NS), based on the combination of the findings.

Finally, the categories were grouped to synthesize the evidence found, and the ConQual approach was used to assess studies ^17^. The recommendations were formulated based on the syntheses and were also assessed according to the JBI guidelines ^25^.

## Results

**Figure 1:**
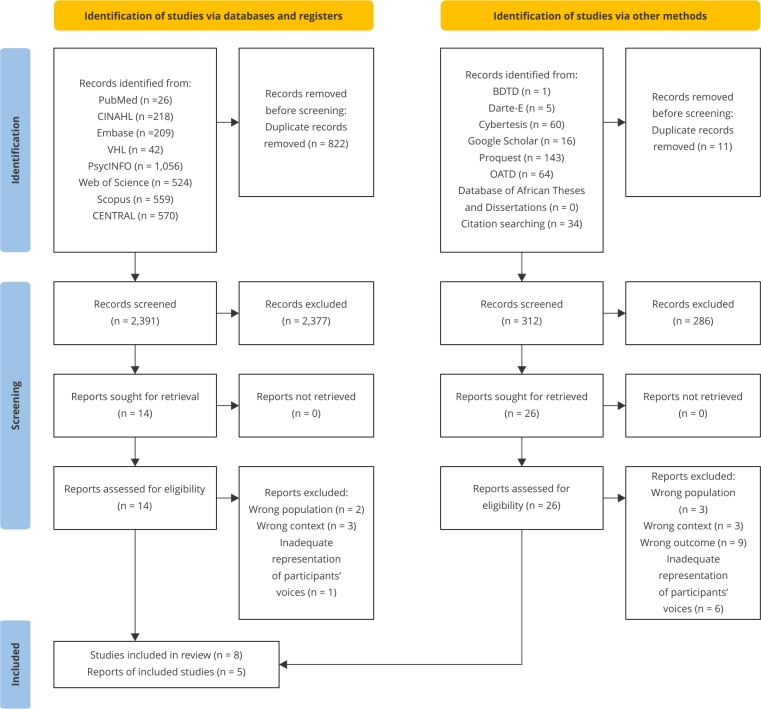
*Preferred Reporting Items for Systematic Reviews and Meta-Analyses* (PRISMA) diagram of the study selection process.

BDTD: Brazilian Digital Library of Theses and Dissertations; CENTRAL: Cochrane Central Register of Controlled Trials; CINAHL: Cummulative Index to Nursing & Allied Health Literature; DART-e: DART-Europe E-theses Portal; OATD: Open Access Theses and Dissertation; VHL: Virtual Health Library.

In total, 3.527 studies were retrieved. From these, 833 were excluded as duplicates, and 2.694 studies were sent to read the titles and abstracts by two independent reviewers, resulting in 40 studies receiving full reading. At this step, five studies were excluded due to not meeting the population criteria, six due to context, nine due to not presenting the perceptions of people involved in the mental health care of children and adolescents, and seven due to not presenting the speech of study participants. In the end, 13 studies were included ^12^ ^,^ ^15^ ^,^ ^26^ ^,^ ^27^ ^,^ ^28^ ^,^ ^29^ ^,^ ^30^ ^,^ ^31^ ^,^ ^32^ ^,^ ^33^ ^,^ ^34^ ^,^ ^35^ ^,^ ^36^, as presented in the flow diagram PRISMA (Figure 1).

Out of the 13 analyzed studies in this review, only one was published before 2011 ^36^, eight were published from 2011 to 2019 ^15^ ^,^ ^28^ ^,^ ^29^ ^,^ ^30^ ^,^ ^31^ ^,^ ^32^ ^,^ ^33^ ^,^ ^34^ and four were published from 2020 to 2022 ^12^ ^,^ ^26^ ^,^ ^27^ ^,^ ^35^. Geographically, studies covered all continents except Africa, with the most conducted in America ^12^ ^,^ ^30^ ^,^ ^32^ ^,^ ^33^ ^,^ ^36^, followed by Oceania ^15^ ^,^ ^31^ ^,^ ^34^ ^,^ ^35^, Europe ^26^ ^,^ ^28^ ^,^ ^29^, and Asia ^27^. Most studies focused on adolescent users as participants ^15^ ^,^ ^28^ ^,^ ^29^ ^,^ ^30^ ^,^ ^31^ ^,^ ^32^ ^,^ ^33^ ^,^ ^34^ ^,^ ^35^, excluding children. Some studies interviewed exclusively the health staff, but some combined interviews with the health staff and family members ^12^ ^,^ ^27^. Studies were predominantly (77%) conducted in hospital inpatient units ^15^ ^,^ ^27^ ^,^ ^28^ ^,^ ^29^ ^,^ ^30^ ^,^ ^31^ ^,^ ^32^ ^,^ ^34^ ^,^ ^35^ ^,^ ^36^, and the study samples varied from five to 82 participants. Box 1 shows the details about the features of the included studies.

**Box 1:** Features of the included studies (n = 13).

STUDY (YEAR)	COUNTRY	INTERVIEWED POPULATION	SEX	AGE (YEARS)	SAMPLE SIZE	STUDY CONTEXT
Bjønness et al. ^26^ (2022)	Norway	Family members	Female and male	Do not apply	12	Community mental health services
Fu et al. ^27^ (2021)	China	Staff and family members	Female and male	Do not apply	34 and 15	Hospital inpatient unit
Gill et al. ^28^ (2016)	United Kingdom	Adolescents	Female and male	14-17	12	Hospital inpatient unit
Haynes et al. ^29^ (2011)	United Kingdom	Adolescents	Female and male	13-19	10	Hospital inpatient unit
Moses ^30^ (2011)	United States	Adolescents	Female and male	13-18	82	Hospital inpatient unit
Moura & Matsukara ^12^ (2022)	Brazil	Staff and family members	Female and male	Do not apply	6 and 12	Community mental health service
Patterson et al. ^31^ (2015)	Australia	Adolescents	Female and male	13-17	43	Hospital inpatient unit
Rosado ^32^ (2019)	United States	Adolescents	Female	12-17	14	Hospital inpatient unit
Rossi et al. ^33^ (2019)	Brazil	Adolescents	Female and male	16-17	5	Community mental health service
Salamone-Violi et al. ^34^ (2015)	Australia	Adolescents	Female and male	15-17	11	Hospital inpatient unit
Stanton et al. ^35^ (2020)	New Zealand	Adolescents	Female and male	12-20	15	Hospital inpatient unit
Thabrew et al. ^15^ (2020)	New Zealand	Adolescents	Female	15-17	9	Hospital inpatient unit
Walter et al. ^36^ (2006)	United States	Family members	Female and male	Do not apply	14	Hospital inpatient unit

Source: prepared by the authors.

### Studies methodological quality

Regarding methodological quality, no study presented less than 80% quality. Thus, this review presents a high degree of reliability, according to the JBI ConQual method ^17^, in all syntheses prepared. Box 2 details information on the methodological quality of each study.

**Box 2:** Methodological assessment of studies.

STUDY	QUESTIONS										TOTAL (%)
	1	2	3	4	5	6	7	8	9	10	
Bjønness et al. ^26^	Yes	Yes	Yes	Yes	Yes	Yes	Yes	Yes	Yes	Yes	100
Fu et al. ^27^	Yes	Yes	Yes	Yes	Yes	Yes	Yes	Yes	Yes	Yes	100
Gill et al. ^28^	Yes	Yes	Yes	Yes	Yes	Yes	Yes	Yes	Yes	Yes	100
Haynes et al. ^29^	Yes	Yes	Yes	Yes	Yes	Yes	Yes	Yes	Yes	Yes	100
Moses ^30^	Yes	Yes	Yes	Yes	Yes	Yes	No	Yes	Yes	Yes	90
Moura & Matsukara ^12^	Yes	Yes	Yes	Yes	Yes	Yes	No	Yes	Yes	Yes	90
Patterson et al. ^31^	Yes	Yes	Yes	Yes	Yes	Yes	Yes	Yes	Yes	Yes	100
Rosado ^32^	Yes	Yes	Yes	Yes	Yes	Yes	Yes	Yes	Yes	Yes	100
Rossi et al. ^33^	Yes	Yes	Yes	Yes	Yes	Yes	No	Yes	Yes	Yes	90
Salamone-Violi et al. ^34^	Yes	Yes	Yes	Yes	Yes	Yes	Yes	Yes	Yes	Yes	100
Stanton et al. ^35^	Yes	Yes	Yes	Yes	Yes	Yes	No	Yes	Yes	No	80
Thabrew et al. ^15^	Yes	Yes	Yes	Yes	Yes	Yes	Yes	Yes	Yes	Yes	100
Walter et al. ^36^	Yes	Yes	Yes	Yes	Yes	Yes	No	Yes	No	Yes	80
Total (%)	100	100	100	100	100	100	61	100	92	92	

Source: prepared by the authors, based on the *JBI Critical Appraisal Skills Program Qualitative Research Checklist* ^17^.

Questions: (1) Is there congruity between the stated philosophical perspective and the research methodology?; (2) Is there congruity between the research methodology and the research question or objectives?; (3) Is there congruity between the research methodology and the methods used to collect data?; (4) Is there congruity between the research methodology and the representation and analysis of data?; (5) Is there congruity between the research methodology and the interpretation of results?; (6) Is there a statement locating the researcher culturally or theoretically?; (7) Is the influence of the researcher on the research, and vice- versa, addressed?; (8) Are participants, and their voices, adequately represented?; (9) Is the research ethical according to current criteria or, for recent studies, and is there evidence of ethical approval by an appropriate body? (10) Do the conclusions drawn in the research report flow from the analysis, or interpretation, of the data?

### Categories and syntheses

Following the JBI methodology for qualitative systematic reviews ^17^, 169 findings and illustrations were found. All of them are available in the Supplementary Material (Box S2; https://cadernos.ensp.fiocruz.br/static//arquivo/suppl-e00016324_6066.pdf). Almost a third of the findings (53) are about procedures; another 37 findings referred to the importance of relationships during crisis intervention; 37 were about the positive emotional responses to treatment; and 28 were about the negative emotional responses to treatment . Finally, 14 findings showed the perceptions of users, family members, and staff about the difficulties and strengths of child and adolescent mental health staff and services. All these findings were grouped into 29 categories, and five syntheses of evidence, as shown in Box 3.

**Box 3:** Findings and illustrations per category, synthesis, credibility, and dependability.

FINDINGS AND ILLUSTRATIONS	CATEGORY	SYNTHESIS	DEPENDABILITY	CREDIBILITY	ConQual SCORE ^17^
8U	Relationship with peers	Importance of relationship during treatment	High	Total: 37 findings (36U + 1C)	Moderate
9U	Relationship with health staff				
10U	Family support				
6U	Communication between staff, family members and users				
1C + 3U	Family role during treatment				
6U	Individual therapy	Importance of procedures during treatment	High	Total: 53 findings (51U + 2C)	Moderate
1C + 3U	Group therapy				
1C + 6U	Personalized treatment				
22U	Power of music therapy				
3U	Psychotropic medication				
3U	Family intervention				
4U	Restraint				
4U	Community mental health service treatment				
5U	Lack of privacy	Negative emotional responses to treatment	High	Total: 28 findings (28U)	High
6U	Stigma				
8U	Exclusion of daily life				
5U	Hospital routine				
3U	Distance from beloved ones				
1U	Fear of returning to the inpatient unit				
1C + 6U	Coping skills	Positive emotional responses to treatment	High	Total: 37 findings (35U + 2C)	Moderate
5U	Discharge planning				
7U	Acceptance				
1C + 7U	Adolescents’ decision-making				
6U	Treatment outcomes				
4U	Sense of security				
2U	Improvement of labor process	Issues about health staff and health services	High	Total: 14 findings (14U)	High
4U	Relationship between family members and health services				
4U	Emergency as mental health service				
4U	Assessment of the staff				

C: credible; U: unequivocal.

A large numerical difference between hospital services (n = 10) and community services (n = 3) was found in the sample, hindering the comparison betweeen the models of care in each type of service, as many categories were created based on the findings only from studies on hospital services due to greater data availability in absolute numbers. However, it is noteworthy that despite this difference, out of the 29 categories, 12 were formed by findings from studies on both services (individual therapy; family support; personalized treatment; communication between staff, family members, and users; relationship between family members and health services; coping skills; psychotropic medication; family intervention; restraint; acceptance; adolescents’ decision-making; emergency as mental health service) and two were made up exclusively by findings from studies of community services (family role during treatment; community mental health service treatment).

Following, the five syntheses will be presented together with the categories that were grouped for their formation.

#### Synthesis 1 - Importance of relationship during treatment

Relationships were relevant aspects of treatment, regardless of whether they were evaluated as positive or negative. Family members emphasized the importance and need to also be taken care of so that they can help and improve the care and relationship with their children.

Regarding “relationships with peers” ^15^ ^,^ ^28^ ^,^ ^29^ ^,^ ^30^ ^,^ ^34^ ^,^ ^35^, adolescents valued sharing care experiences, feeling validation, belonging, and support. Few reported negative experiences, highlighting the importance of peers.

“*It’s the other kids that make it work; that gives you hope*” ^34^ (p. 573).

Regarding “relationships with health staff” ^15^ ^,^ ^28^ ^,^ ^30^ ^,^ ^34^, adolescents considered the staff as essential in mental healthcare, feeling supported and listened to. The staff was seen as a substitute family, but some attitudes were perceived negatively, as unfair or invasive, and a strong attachment to the staff made it difficult to return home.

“*You can talk to them about anything, and, like, they didn’t judge you about it*” ^30^ (p. 126).

Regarding “family support” ^12^ ^,^ ^26^ ^,^ ^27^ ^,^ ^34^ ^,^ ^35^ ^,^ ^36^, family members reported the desire to be advised on how to deal with their children’s behavior. Crises revealed strengths and weaknesses that some families had. A good assessment was found for family members who were monitored by the reference professional in their children's case.

“*I couldn’t handle my kids, finances, housework, cooking, shopping... I couldn’t take care of anything.* [Case management] *pretty much was the only thing holding my family together*” ^36^ (p. 617).

Regarding “communication between staff, family members, and users” ^26^ ^,^ ^27^ ^,^ ^30^ ^,^ ^34^ ^,^ ^36^, family members complained about the lack of information at different times during their children’s treatment, which hindered aiding and contributing to the adolescents’ care, as well as better understanding their child’s condition and taking more effective care actions. The staff, on the other hand, felt less confident and had difficulty communicating effectively with family members. Adolescents reported communication as a positive aspect when they were able to name what they felt and the meaning of their condition. They understood communication as something negative when they found themselves in an unfamiliar place and did not have enough information during hospitalization.

“*I mean, I didn’t even know where I was, and then I was expected to just go to sleep and speak to someone in the morning. I thought someone would have explained more then. I didn’t even know who it was in the other bed; it was weird*” ^34^ (p. 574).

Regarding “family role during treatment” ^26^ ^,^ ^33^, family members emphasized their supportive role with their children during treatment and adolescents understood the importance of this, which provided feelings of security and trust. Family members also sought out professionals and communicated with them what they considered to be appropriated for treatment and measures for their children.

“*...to talk about what helped me the most... I think about my mother a lot. Because when I told her about what was happening, she really understood me and tried to help me... she didn't judge me...*” ^33^ (p. 6).

#### Synthesis 2 - Importance of procedures during treatment

Most procedures had good results from the perspective of those involved.

Regarding “individual therapy” ^15^ ^,^ ^27^ ^,^ ^30^ ^,^ ^33^ ^,^ ^34^, both family members and users perceived less access than necessary, and the users said they preferred individual interventions to collective interventions.

“*I wasn’t getting very much one-to-one time, which was important to me and I know it was important to other people there too*” ^30^ (p. 131).

Regarding “group therapy” ^27^ ^,^ ^30^ ^,^ ^34^, adolescents’ perceptions were divided. Some referred to the groups as uncomfortable and inefficient spaces. They also felt that staff were too passive and analytical in this context and described groups as unsafe places to share information. However, some adolescents had opposite perceptions and perceived the groups as one of the aspects that helped most during hospitalization, understanding the space as powerful for their care. It is noteworthy that this category was created only with findings from studies carried out in hospital services, which provides a snapshot of adolescents’ opinions only related to group therapy in this specific setting.

“*Group was just good ‘cause we all, like, opened up. And a lot of people ended up crying. And we just, like, got through, like, what happened to us. And, like, it just, it was kind of, like, a relief to get it off your chest and, like, finally open up to somebody that, like, as open as we were*” ^30^ (p. 127).

Regarding “personalized treatment” ^15^ ^,^ ^26^ ^,^ ^27^ ^,^ ^30^ ^,^ ^36^, family members asked for personalized care for their children, so that it was effective and meaningful. Adolescents also wanted specific answers to their needs.

“*We often experience that they try to use one success story and put it on a second child without asking. Observe, explore what’s right for them. It is time-consuming and more expensive, but you get poor results if you do hasty work*” ^26^ (p. 1000).

Regarding “power of music therapy” ^31^ ^,^ ^32^, music therapy was perceived as a positive intervention during hospitalization by users and the team. Most users perceived the strategy as a good way to communicate their feelings.

“*Coming out of* [music therapy]*, I felt like the voices in my head were gone. I wanted to feel better and I came out of there with a purpose, knowing that things like that were going to help me in my journey*” ^32^ (p. 136).

Regarding “psychotropic medication” ^12^ ^,^ ^30^ ^,^ ^33^, few adolescents identified it as the most important benefit of hospitalization. In community mental health services, medication use was highlighted as a strategy for crisis situations. Staff also discussed with adolescents about the continuation and whether or not it was necessary in their treatment.

“*I keep going* [to the Psychosocial Care Center]*, but I don’t think I need medication... I think I take too much medication, it makes me very sleepy...*” ^33^ (p. 7).

Regarding “family intervention” ^26^ ^,^ ^30^ ^,^ ^34^, both family members and adolescents consider this type of care to be important and strategic. However, some adolescents reported that the family interventions were of little use. They also mentioned feeling unheard and perceived that the professionals were against them and in favor of the family members.

“*It has been a long process to understand what the disease entails. I feel that being part of a family group helped to get some of those answers. Together with other families in the same or similar situations, we could share experiences*” ^26^ (p. 1000).

Regarding “restraint” ^12^ ^,^ ^30^ ^,^ ^34^, some adolescents reported as a positive fact that they had limited physical space in the hospital - such as their own room or the hospital wing -, as it offered them the opportunity to be cared for by others, thus not having to worry about having to take care of themselves. However, adolescents also reported being scared when they saw peers being physically or mechanically restrained by staff. Family members realized that community services used restraint only in cases of extreme need for crisis intervention and emphasized that this strategy also causes distress to families.

“*Once, he cut glass with his hands and got very upset, they took him to a room and held him, the technician said ‘You can go away, he’ll be fine, we’ll talk to him!’ I left, but on the other side of the street I heard his screams. That day I left very depressed. So, I called and she said ‘He’s fine now! He’s playing and he has already eaten! He’s no longer in crisis’*” ^12^ (p. 14).

Regarding “community mental health service treatment” ^12^, professionals and family members reported that specific strategies, such as daytime hospitality and user embracement, were important in managing crisis situations.

“*They helped me a lot... he stayed here, they took care of him and gave him a lot of attention*” ^12^ (p. 9).

#### Synthesis 3 - Negative emotional responses to treatment

For adolescents, hospitalization triggered negative feelings related to the distance from everyday life, hospital rules and third parties’ perception of their mental condition.

Regarding “stigma” ^27^ ^,^ ^28^ ^,^ ^29^ ^,^ ^30^ ^,^ ^34^, before hospitalization, adolescents had stigmatized views on mental health but this perception changed after the experience. Adolescents feared facing stigma and deteriorating friendships after discharge.

“*Like, ‘cause it’s called a mental hospital and that kinda makes you feel like if you’re there, you must be mental, you must be*” ^30^ (p. 132).

Regarding “lack of privacy” ^15^ ^,^ ^28^ ^,^ ^30^ ^,^ ^34^, users complained about the lack of privacy and restriction of personal freedom during hospitalization as they perceived the team to be very invasive at times.

“*No one has time to spend alone, because as you can see all these doors are locked. You can’t go into your bedroom... the only place you could possibly have all by yourself is the loo*” ^28^ (p. 60).

Regarding “exclusion of daily life” ^15^ ^,^ ^28^ ^,^ ^29^ ^,^ ^30^ ^,^ ^34^ ^,^ ^35^, adolescents reported that during their hospitalization they felt as if they were in a parallel reality, an environment extremely different from home or the outside world. The inpatient unit was seen as an interruption to everyday life, which caused them to miss important events and valuable aspects of their lives. They also reported that participating in everyday activities, even within the hospital, was important to recreate a familiar reality in an unfamiliar context. At the same time, having time to reflect on their issues and life circumstances without the daily pressures of home was a useful aspect of the brief hospitalization for them.

“*It’s frustrating being locked up, being restricted to a lot of things... missing out on life, not experiencing what a normal teenager should experience*” ^35^ (p. 8).

Regarding “hospital routine” ^28^ ^,^ ^29^ ^,^ ^30^, adolescents complained about having to follow it, being forced to participate in scheduled activities and not being able to choose what to do. For them, restrictions on verbal and physical contact with other adolescents were particularly difficult, as these interactions were seen as comforting and with the potential for care. However, the users also said that having a routine was important to create inner peace and distract from their issues.

“*I got quite violent, um towards the staff because I saw them as keeping me prisoner, keeping me prisoner inside this place... I just felt really hostile towards the staff for keeping me here, for locking me in*” ^29^ (p. 152).

Regarding “distance from beloved ones” ^29^ ^,^ ^30^, adolescents expressed feelings of isolation, loneliness, stress, and anxiety due to being away from home and disconnected from family and friends.

“*Every single day my sister is asking for me to come and see her, but she just doesn’t realize I can’t come see her... I was the man of the family, the very time the family needed me I was locked up*” ^29^ (p. 152).

Regarding “fear of returning to the inpatient unit” ^27^, users referred that coming back to inpatient unit would mean a personal failure.

“*Back to square one*” ^28^ (p. 62).

#### Synthesis 4 - Positive emotional responses to treatment

Adolescents experienced positive feelings of self-knowledge, plans and autonomy in self- care.

Regarding “coping skills” ^28^ ^,^ ^29^ ^,^ ^30^ ^,^ ^33^, it was shown that, in crisis situations caused by despair, anguish, inferiority, and abandonment, adolescents had difficulty developing coping strategies and placed themselves in risky situations. During treatment, the adolescents reported that they were able to better understand their difficulties and create cognitive and behavioral strategies to deal with critical situations.

“*All these little things all kind of add up to one big change... I feel like I've been transformed in a way, like upgraded to a new me*” ^28^ (p. 61).

Regarding “discharge planning” ^15^ ^,^ ^28^ ^,^ ^29^ ^,^ ^34^, the adolescents spoke about the importance of having a transition between hospitalization and returning home, before being discharged. They reported that, after discharge, they knew they needed a support network, and continuity in mental health care and expressed the desire to be seen as “normal” by others. Thinking positive and knowing that they could resume what they had to pause during hospitalization helped them while they were hospitalized.

“*Maybe during that time I would try having some leave, see if it’s ok, then have more leave, go see some friends, do what I do normally, maybe stay overnight and then go after that*” ^34^ (p. 574).

Regarding “acceptance” ^15^ ^,^ ^27^ ^,^ ^33^ ^,^ ^34^ ^,^ ^35^, it was presented that accepting your mental health issue and accepting help was a debated subject by adolescents.

“*I dunno if I was hoping it would happen, I just, well, I just wanted to feel better and I needed help, so yeah, I guess it was alright*” ^34^ (p. 571).

Regarding “adolescents’ decision-making” ^15^ ^,^ ^26^ ^,^ ^30^ ^,^ ^35^, the adolescents mentioned their desire to be listened to about their treatment and reported several situations in which they were not listened to or did not have their wishes respected. Users and family members said that, without being committed to the treatment, the interventions would not be effective. The treatment provided them an opportunity to be more responsible about their lives. Family members also spoke about the dilemma of how to empower their children in this process. This category included findings from one study in community services and the others in hospital services, which may be related to the struggle of adolescents to be heard and participate in the process.

“*I would definitely have appreciated being included more. Maybe asked; I mean, I don’t really know how to fix the problem, I just know that I didn’t appreciate being told what to do and not being included*” ^15^ (p. 3).

Regarding “treatment outcomes” ^27^ ^,^ ^28^ ^,^ ^30^ ^,^ ^34^ ^,^ ^35^, several family members noticed behavioral changes in their children and thought this was due to the treatment. The adolescents reported important improvements, such as being calmer, more confident, and healthier, but they realized that they still had difficulties and planned to improve little by little. Some adolescents said they were angry and disappointed because they thought they were discharged before they were ready.

“*I feel significantly more confident than I did before coming in here. I still feel emotional, sad, and anxious but I feel like I’ve learned a lot of things and lessons here that over time I will be applying that will help me as an individual just cool off and be a generally healthier person*” ^35^ (p. 10).

Regarding “sense of security” ^15^ ^,^ ^28^ ^,^ ^30^, adolescents reported about feeling safe during hospitalization but some said that this could also be a negative aspect since this does not happen in the real world, where there are difficulties and they are not prepared. Some users said they were anxious and scared in the hospital environment.

“*We’re being watched quite a lot of the time... I think it’s quite good because I don’t have a chance to hurt myself and I know I’m safe*” ^28^ (p. 61).

#### Synthesis 5 - Issues about health staff and health services

Mental health professionals and health services were assessed as lacking in some aspects of care, and improvements were highlighted by the staff, users, and family members.

Regarding “improvement of labor process”, professionals suggested increasing the staff and optimizing the division of labor. They also spoke about the need for supervision and training to deal with some mental health problems, such as adolescents attempting suicide.

“*We do not have much time to communicate with patients and solve some of their psychological problems. Understaffing is a factor, and the second may be the division of labor is not optimized...*” ^27^ (p. 5).

Regarding the “relationship between family members and health services” ^12^ ^,^ ^26^ ^,^ ^33^ ^,^ ^36^, several parents reported that they had to coordinate services for their children’s care. They also complained about barriers to accessing mental healthcare services and the lack of communication between services. On the other hand, some reported trusting and having a good relationship with the services. Adolescents said mental health services are part of their support network.

“*We had to be the mediator between them (different healthcare services) on things they ought to know. It is silly because it takes a lot of energy, and it has taken a long time for us to understand the system. Who is responsible, and who should take the initiative? In the end, we have to do it*” ^26^ (p. 1000).

Regarding “emergency as mental health service” ^12^ ^,^ ^36^, family members reported difficulties in getting support in emergency units when their children were in crisis and talked about the delay and bureaucracy in the process of admission to the service.

“*We were* [at the hospital ER] *for like about an hour and a half before* [the screener] *even showed up because nobody had informed him that we were there yet. Since we had to go through the emergency room, there was a long wait in the emergency room. We went up there at, it was between 8:00 and 9:00 a.m. And we didn’t get out of there until about 2:00 or 3:00 in the afternoon*” ^36^ (p. 617).

Regarding “assessment of the staff” ^15^ ^,^ ^27^, adolescents perceived the team as unprepared to deal with mental health problems. Professionals said they had low expectations about what they could do and that working with adolescents with suicidal behavior requires some personal characteristics, such as patience and communication, and they need certain professional skills to be trained.

“*We have to learn communication skills, and the learn some psychological counseling methods to empathize with patients, which is very difficult to learn...*” ^27^ (p. 4).

Due to the high methodological quality of the studies, the dependability of the syntheses was also classified as high. The credibility of the syntheses varied from high to moderate, depending on the assessment of the findings and illustrations. It means that most of the findings were considered unequivocal (findings accompanied by an illustration that is beyond reasonable doubt and thus not open to challenge) and credible (findings accompanied by an illustration lacking clear association with it and therefore open to challenge).

## Discussion

### Importance of relationships during treatment

Interpersonal relationships are fundamental to the support and mental health of children and adolescents ^37^. Considering these benefits, the Pan American Health Organization (PAHO) has encouraged the use of “peer support” as a powerful tool for care, rapprochement, and support in mental health services ^38^. Peer support considers that the meeting between people who are going through or have gone through similar situations promotes understanding, exchange of experiences, and acceptance of the suffering experienced without judgment ^39^ ^,^ ^40^.

In addition to peer support, relationships between users and staff, inherent to mental health care, and the care of children and adolescents during times of crisis, also play an important role during the care process. A good relationship between health professionals and users favors the achievement of faster, more efficient results and a better prognosis ^41^. For mental health staff, establishing and maintaining good communication with family members and users is essential, as it directly impacts treatment ^42^. However, it can be a challenge, as the complexity of cases, including crisis situations, shows an emotional impact on these professionals, which can cause intense strain on these relationships ^43^. As a result, users may feel that they are not being heard or welcomed ^15^ ^,^ ^30^ ^,^ ^35^, at the same time that the team itself may experience difficulty establishing a good relationship with adequate communication ^27^ ^,^ ^34^ ^,^ ^36^ ^,^ ^41^.

As for the family, they may feel that they lack information to better care for their child or adolescent and feel helpless ^36^ ^,^ ^44^. Therefore, it is common for family members to feel disoriented and, as a result, ask for more support and guidance to know what to do and how to act with their children who are in this period of more intensive care ^13^ ^,^ ^26^ ^,^ ^27^ ^,^ ^34^ ^,^ ^36^. A study showed that family members’ capacity to seek and feel capable of care intensifies once both family members and users understand and value the relevant support role that the family has during the care process ^45^.

### Importance of procedures during treatment

Relationships in the care process occur via casual, individual, or group meetings. According to Lima et al. ^46^, the space for individual therapeutic care, combined with the bond between user and professional, creates an environment conducive to expressing feelings and experiences, including those that led to the crisis. Group therapy is also valued for the exchange of similar experiences, especially in crises ^46^ ^,^ ^47^. Personalized treatment, respecting the uniqueness of each individual, is fundamental in mental health services, such as in Psychosocial Care Centers, with the Singular Therapeutic Project (STP) ^48^. STP enables planning the treatment considering individual experiences, aligning and comanaging the development of care between mental healthcare professionals, users, and family members ^49^, which are reevaluated in a crisis situation.

Family support is essential, with an emphasis on listening and welcoming, especially during crises, to identify difficulties and potentialities in family dynamics, which can be both support and triggering factors for crises. This approach expands the assessment of the crisis, identifying the care needed also in family members ^13^ ^,^ ^46^.

In addition to the care provided via relationships, the use of psychiatric medications is commonly employed to deal with crises. However, studies reveal that the numbers referring to the use of psychiatric medication by children and adolescents are increasing worldwide ^50^ ^,^ ^51^ ^,^ ^52^. This can be explained by the current trivialization of psychiatric diagnoses in childhood and adolescence ^53^, despite no evidence being found on the safety and effectiveness of this use in these age groups ^53^ ^,^ ^54^. At the same time, studies point to the negative effects on the global development of children, including socioemotional development ^55^ ^,^ ^56^ ^,^ ^57^.

Several strategies can be used in crisis care and intervention in mental health services, such as music therapy ^31^ ^,^ ^32^ ^,^ ^58^, bodily activities ^59^, and restraint. The latter, according to Moura & Matsukura ^12^, keeps on being a procedure mentioned by family members and mental health staff for crisis intervention in community mental health services for children and adolescents in Brazil as the last possible option to deal with a crisis. However, the use of restraint, especially physical, mechanical, or chemical, is still quite controversial. Perers et al. ^60^ emphasizes that several strategies can be used as alternatives to the use of restraint, such as child-centered initiatives and behavioral management strategies. In addition to being very well evaluated, they should be prioritized in the mental health care of children and adolescents as child- and family-centered care initiatives ^60^.

### Positive and negative emotional responses to treatment

Mental health crises are marked by high fragility and suffering ^61^. During treatment, adolescents recognize positive factors and develop coping strategies, benefiting from environments that promote decision-making and protagonism ^28^ ^,^ ^29^ ^,^ ^30^ ^,^ ^33^ ^,^ ^62^. They notice improvements in calmness and abilities to deal with difficulties, plan for discharge, and return to routine ^29^ ^,^ ^63^ ^,^ ^64^.

However, treatment still faces the stigma of mental illness ^2^ ^,^ ^65^, challenges as distance from family members and friends ^29^ ^,^ ^30^, lack of privacy ^15^ ^,^ ^29^ ^,^ ^30^ ^,^ ^34^, and adaptation to hospital routine ^28^ ^,^ ^29^ ^,^ ^30^. Exclusion from everyday life is especially difficult for school-age adolescents, hindering future life planning ^15^ ^,^ ^28^ ^,^ ^29^ ^,^ ^30^ ^,^ ^34^ ^,^ ^35^. Crisis care is proposed in open and community environments, which rethink care strategies and take advantage of the transformative potential of crises ^66^. The effort of public authorities and health services to offer a model of mental care for children and adolescents that avoids punitive and exclusionary logic is crucial ^67^.

### Issues about health staff and health services

During the crisis, caregivers of children and adolescents criticized the delay in initial care ^12^ ^,^ ^36^ ^,^ ^68^ and the disorganization of mental health services ^36^ ^,^ ^69^. Moreover, they faced the inadequacy of services to the specific needs of their children ^12^. The importance of health systems organizing care that promptly and effectively meets mental health needs in crises is highlighted ^48^. Health professionals recognize the need to improve work processes to adequately serve and care for users and families ^27^. On the other hand, users, whether adolescents, family members, or caregivers, expect staff to be well prepared for intervention and care ^15^ ^,^ ^70^.

### Recommendations for practice, public policies, and research

For clinical practice, it is essential to invest in interpersonal relationships during interventions. Individualized care is recommended, meeting the specific needs of each case. Rigid and invasive procedures, such as restraint, should be avoided due to potential harm. Professionals must be trained to deal with the complexities of crises, provide shared care, and promote the protagonism of users and families.

For public policies, it is crucial to invest in the qualification of professionals and in the strengthening of support and care networks. It is also recommended to invest in strategies to minimize the damage caused by hospitalization, as seen in studies. Investing in 24-hour hospitality in community mental health services for children and adolescents could be promising, as they are longitudinal healthcare services with reference staff for users and family members, and can work as part of a care network in the territory to make the return to daily life as quickly and easily as possible.

In the field of research, studies are suggested on the experiences of children and adolescents in community mental health services and on the potential and difficulties in training child and adolescent mental health teams.

According to the *JBI Feasible, Appropriate, Meaningful, and Effective* (FAME) scale, all of these recommendations are grade A, indicating strong evidence of benefits, quality of evidence, and consideration of users’ values and experiences ^71^.

### Limitations

As a limitation, this review did not include studies about childhood or listening to children who received interventions in crises. Few studies described intervention in child and adolescent crises in community mental health services, which limited the analysis mainly to hospital experiences. A scoping review ^72^ published in 2023 that provided a comprehensive overview of existing and upcoming community mental healthcare approaches concluded that less than half of the included papers are empirical studies, and a large part of the included papers were composed of descriptive or opinion papers. The authors suggested more empirical research on this subject.

## Conclusion

This review found that the perceptions of mental healthcare professionals, family members, and users about children and adolescents’ mental health crisis intervention at hospitals and community mental health services could be categorized into procedures; importance of relationships during crisis intervention; positive and negative emotional responses to treatment; and perceptions of users, family members, and staff about the difficulties and strengths of child and adolescent mental health staff and services. It was possible to observe convergent perceptions about interventions in crises experienced by children and adolescents in mental health services. Professionals pointed to the need to improve the labor process and the staff itself to raise the level of care. Family members highlighted the same needs in addition to recognizing that they need to be more responsible for their children’s care. Adolescents perceived negative and positive aspects of care in different environments, also suggesting ways to improve the care they undergo.

Further research should be developed addressesing this topic in community mental health services, given that most of the studies that were part of this review were conducted in a hospital context. It is also suggested to carry out research that seeks to listen to the perceptions of children, as understanding perceptions is one of the paths towards improving care and, consequently, improving users’ experiences at this time of suffering.
