# Supplementary material for: Perceptions about children and adolescents’ mental health crisis intervention: a qualitative systematic review
Source: Cad Saude Publica. 2024 Dec 20;40(11):e00016324. doi: 10.1590/0102-311XEN016324 (PMC11654114; doi:10.1590/0102-311XEN016324)
Supplement: Supplementary file 1 [file 1678-4464-csp-40-11-EN016324-s.pdf]

SUPPLEMENTARY MATERIAL

Box S1 Search strategies for databases.

Search strategy for MeSH terms databases	("crisis intervention"[MeSH Terms] OR "crisis interventions"[Title/Abstract] OR "brief advice"[Title/Abstract] OR "screening and brief intervention"[Title/Abstract] OR "brief treatment"[Title/Abstract] OR "brief treatments"[Title/Abstract] OR "brief interventions"[Title/Abstract] OR "brief intervention"[Title/Abstract] OR "critical incident stress debriefing"[Title/Abstract]) AND ("adolescent"[MeSH Terms] OR "adolescents"[Title/Abstract] OR "adolescence"[Title/Abstract] OR "teens"[Title/Abstract] OR "teen"[Title/Abstract] OR "teenagers"[Title/Abstract] OR "teenager"[Title/Abstract] OR "youth"[Title/Abstract] OR "youths"[Title/Abstract] OR "child"[MeSH Terms] OR "children"[Title/Abstract]) AND ("mental health"[MeSH Terms] OR "mental health assistance"[Title/Abstract]) AND ("hospitals"[MeSH Terms] OR "hospital care"[Title/Abstract] OR "packaged hospital"[Title/Abstract] OR "packaged hospitals"[Title/Abstract] OR "general hospital"[Title/Abstract] OR "general hospitals"[Title/Abstract] OR "mental health services"[MeSH Terms] OR "mental hygiene services"[Title/Abstract] OR "mental hygiene service"[Title/Abstract] OR "mental health service"[Title/Abstract])
Search strategy for DeCS terms databases	(“intervenção na crise” OR “conselho breve” OR “interrogatório em incidente crítico com estresse” OR “intervenções breves” OR “tratamento breve” OR “triagem e intervenção breve” OR “intervención en la crisis”) AND (hospitais OR “assistência hospitalar” OR “atención hospitalaria” OR “centro hospitalar OR “centros hospitalares” OR hospital OR nosocômio OR nosocômios OR “hospitais de emergência” OR “hospital de emergência” OR “hospitais gerais” OR “hospital geral” OR “hospitales generales” OR “hospitales de urgencia” OR hospitales OR “serviços de saúde mental” OR “centro de atendimento psicossocial” OR “centros de atendimento psicossocial” OR “centros de atenção psicossocial” OR “núcleos de atenção psicossocial” OR “serviço residencial terapêutico em saúde mental” OR “serviços de higiene mental” OR “servicios de salud mental”) AND (adolescente OR adolescência OR adolescentes OR jovem OR jovens OR juventude OR criança OR crianças OR niño) AND (“assistência à saúde mental” OR “assistência em saúde mental” OR “atención a la salud mental” OR “saúde mental’ OR “área de saúde mental” OR “salud mental”)
Other databases	Adaptations were made using this review descriptors.

Box S2 Findings and illustrations of review.

Category "Relationship with peers"			
		Study	Assessment*
Finding 1	Having a shared experience with fellow inpatients led to a sense of validation and belonging.	Gill et. al[1]	U
Illustration 1	"When I talk to [a fellow inpatient] about my experiences I feel that they know what I'm talking about and that they've been through a similar sort of situation"		
Finding 2	Participants relationships with other inpatients were characterised by an ongoing process of learning to deal with others distress.	Haynes et. al[2]	U
Illustration 2	"Kirsty and Hannah they wouldn't eat...Dont know what was the matter with them, think they had some sort of eating problem... I felt really awful, there I am eating and those two are sitting there just picking at their food, while I was sitting there eating my chips and everything"		
Finding 3	Participants described developing supportive (i.e. reciprocal and non-judgmental) relationships with other young inpatients, which appeared to help them cope with the negative emotions associated with hospitalization, often through the simple process of listening and talking	Haynes et. al[2]	U
Illustration 3	"If they're wanting to kick off then I'll sit there and talk to them, make them change their mind. If they want to self-harm I'll sit there and talk to them, stop them from doing it"		
Finding 4	Peers' role in helping refers to the support, companionship and advice they offered.	Moses[3]	U
Illustration 4	"...there was another girl in there that had also tried overdosing on some kind of [medication type]. So it was, like, really similar to what I had done. And it was just reassuring because you're not the only one...Oh, so I'm not just this really messed up girl"		
Finding 5	Finally, a few participants perceived peers at the hospital as negative:	Moses[3]	U
Illustration 5	''And kids don't believe in you and kids just sit there and rag on you, and like a lot of the kids are really mean to you''		
Finding 6	Interaction with others: patients	Salamone-Violi et. al[4]	U
Illustration 6	"It's the other kids that make it work; that gives you hope"		
Finding 7	Contact with peers provided company, understanding and belonging	Stanton et. al[5]	U
Illustration 7	"You can trust them, you can care for them, knowing that they're all going through most likely something like you are"		
Finding 8	A shared journey	Thabrew et. al[6]	U
Illustration 8	"It was quite nice to have other people that kind of understand what you are going through and could relate to what was happening"		

Category "Relationship with health staff"			
		Study	Assessment
Finding 9	They appreciated the staff's persistent approach in supporting them and "not giving up on me"	Gill et. al[1]	U
Illustration 9	I can always talk to someone		
Finding 10	Given the intensity of the inpatient environment and the strong interpersonal bonds that had developed, fellow patients and staff were experienced as akin to a family	Gill et. al[1]	U
Illustration 10	Staff almost become your parents in the sense that they nag you sometimes and you've got to ask permission for things... And the patients almost become like brothers and sisters.		
Finding 11	Several participants, however, also spoke about feeling uncomfortable that they had become "too attached" to the other young people and staff, and worried that this would make it harder for them to leave the inpatient unit and return home to their families	Gill et. al[1]	U
Illustration 11	I don't want to get comfortable because one day I will have to leave... I don't want to be really attached with this place because I will just get disappointed in the end...		
Finding 12	However, for some young people, the inpatient environment was experienced as reinforcing their dependece on others.	Gill et. al[1]	U
Illustration 12	I was quite a independent person... but here you got to rely on people because everything you do has got to be checked with staff members.		
Finding 13	These participants described feeling better as result of interactions with staff who were easy to talk to, good listeners, nonjudgmental, reliable, and supportive:	Moses[3]	U
Illustration 13	You can talk to them about anything, and, like, they didn't judge you about it'		
Finding 14	Staff behavior perceived as unhelpful included: intrusion on space (e.g., ''she	Moses[3]	U

	got right up in my face, not even half a foot away from me'' (001)); meanness, disrespect or impatience: ''a few of them would, like, they would, ike, yell at certain kids, like, like, other teens and stuff, and, you know, like, for something that wasn't necessary.		
Illustration 14	'She didn't really, like, care what I had to say really.		
Finding 15	Also, several youth mentioned that some staff enforced rules unevenly in ways perceived as unfair or arbitrary:	Moses[3]	U
Illustration 15	Yeah, and like somebody tells you one thing and then another person tells you another thing so it's just you don't know what to do because you don't wanna get in trouble for breaking the rule. But then it's just like if somebody told you that you can do it, then you thought you could.		
Finding 16	Interactions with others: staff	Salamone-Violi et. al[4]	U
Illustration 16	Outside of the ward, like if I'm back at school and I've tried to tell my teachers how I'm feeling, they don't understand, they don't really listen, but they do here, they get it.		
Finding 17	Perceived support from some staff	Thabrew et. al[6]	U
Illustration 17	I guess they really understood us, know like how frustrated we were sometimes		

**Category "Family support"**			
		Study	Assessment
Finding 18	The parents also stated a need for support and advice or counselling.	Bjonness et. al[7]	U
Illustration 18	Parental guidance is essential. We realized that here we needed help to be parents. One enters such a mode where we do not have the opportunity to process everything because one does not know what is coming around the next corner.		
Finding 19	Many parents expressed concers regarding how to get along with their children in the future, hoping that psychiatrists or nurses would give them guidance on parenting.	Fu et. al[8]	U
Illustration 19	... I think they need to communicate more with the kids. Psychiatrists and nurses may be more likely to communicate with my daughter than we are. After they know it, they can teach me how to communicate with her...		
Finding 20	Desta maneira, uma situação de crise não revela apenas o sofrimento de uma criança e um adolescente, mas também todas as potências e impotências de sua rede de suporte, dos contextos em que se encontra (ou não) inserido e de suas relações.	Moura & Matsukura[9]	U
Illustration 20	As meninas me passam que a pessoa tem que conversar, quando começa a ficar nessa agitação. [...] Eu fui aprendendo a lidar com ele porque até aí, eu não sabia. Eu só chorava. E aí, eu comecei a me sentir mais forte graças a Deus! Agora quando precisa falar com ele, eu sento e converso [...].		
Finding 21	Advocacy and awareness	Salamone-Violi et. al[4]	U
Illustration 21	It's good that the staff here at least understand and can help my parents understand as well.		
Finding 22	Relatedness with family was supported	Stanton et. al[5]	U
Illustration 22	I think staff's dissection of everything, 'this is what I notice about your son, this is what we've come to a conclusion of', I think that's helped both me and my parents in understanding issues.		
Finding 23	It appeared that the families' definition of a crisis did not match the definition held by responders.	Walter et. al[10]	U
Illustration 23	Well, those people say, "Is he homicidal?" "No." "Is he suicidal?" "No."... "We aren't going to do anything about that." So I always felt like my hands were tied, and I felt stupid most of the time. I didn't know who to turn to, what to do.		
Finding 24	Some parents indicated they wanted coaching about how to handle a child's behaviors, and reported being irritated by hotline responders telling them to call the police.	Walter et. al[10]	U
Illustration 24	Say you call and have been with the [children] all weekend and need help, and they basically relay, "Well, what do you want us to do? Call the police!" I am sorry, I don't want to call the police. They will take a totally different approach. Besides, we have had the police out here before and word in town gets around real fast in this rural community.		
Finding 25	Transportation to the screening location and/or to the hospital was provided by families themselves, by the CMH C staff, by child welfare providers, by secure transport companies, or by police.	Walter et. al[10]	U
Illustration 25	We called them the night before, the crisis team. And they said, "well, we could send the police to pick her up, but you're going to have to find your own way there." And this is like 11:30 at night, you know, half the people in here were already asleep, at least the ones with cars.		

Finding 26	The nine families who received case management felt supported, listened to, and relayed that their children related well with case workers.	Walter et. al[10]	U
Illustration 26	I couldn't handle my kids, finances, housework, cooking, shopping ... I couldn't take care of anything. ... [Case management] pretty much was the only thing holding my family together.		
Finding 27	Interviews reflected a wide array of perspectives on the quality of involved child welfare services ranging from highly satisfied to very unsatisfied.	Walter et. al[10]	U
Illustration 27	I always felt like I could call them [child welfare agency] and ask them questions, or if there was a problem at school, they would be the first people I'd call.		

Category "Communication between staff, family members and users"			
		Study	Assessment
Finding 28	This lack of information limited their ability to contribute to and support the adolescents in decision-making.	Bjonness et. al[7]	U
Illustration 28	It is difficult to advise, recommend something, or push in a direction when one does not know anything about the alternatives. It is challenging to have a dialogue to motivate and accept something when you do not know what the choices consist of. (Parent 3)		
Finding 29	A few participants felt distrustful, making it difficult for them to communicate effectively with patients and gain a deeper understanding of what parents really think.	Fu et. al[8]	U
Illustration 29	" ... They don't want to communicate with people he doesn't trust, so they don't tell us exactly what he thinks. We can't collect all the information, and what we know is one-sided ... " (Psychiatrist, p1)		
Finding 30	Most participants reported that medical staff were busy and communicated little with them and their children; therefore, their understanding of childhood mental health diseases and the treatment mechanisms were limited.	Fu et. al[8]	U
Illustration 30	... I feel like they just come in the morning and ask if they have any questions. No one went to chat with my child or to communicate with her...		
Finding 31	A notable benefit of being hospitalized for 20% of participants involved access to information about a label for their symptoms or the meaning of their illness:	Moses[3]	U
Illustration 31	''I found out how anxious I really am... I thought it was just stress''		
Finding 32	Information	Salamone-Violi et. al[4]	U
Illustration 32	I mean, I didn't even know where I was, and then I was expected to just go to sleep and speak to someone in the morning. I thought someone would have explained more then. I didn't even know who it was in the other bed; it was weird.		
Finding 33	Some families expressed that psychiatrists did not seem to validate their experience or listen to them, that there was a lack of information about medication interactions, and inconsistent or conflicting recommendations from different providers regarding the appropriateness of given medications.	Walter et. al[10]	U
Illustration 33	You know, you've got some doctors that say, "Well, these kids should never be on these meds," and then this doctor here says, "Oh, yeah, we can put him on this, no problem!" ... So who do you believe?		

Category "Communication between staff, family members and users"			
		Study	Assessment
Finding 34	Several parents also looked for suitable therapists and helped convey to them what they considered appropriate treatment and measures for their sons and daughters.	Bjonness et. al[7]	C
Illustration 34	The parents are the ones who know their child best. It is a new health professional who enters. They don't know how this person reacts and what is important for her. So dialogue with parents is essential! And since she doesn't talk much, we have had to tell them what is relevant for them to know. (Parent 6)		
Finding 35	Most importantly, the parents emphasized their supportive role.	Bjonness et. al[7]	U
Illustration 35	The significance of parental support is underestimated. Parents are a resource in a slightly different way. Our resource is to be able to cheer them on, so to speak. 'This was good, so much you have learned, this has done you good'... We have to trust the healthcare services rather than questioning their reliability. I think scepticism closes the doors.		
Finding 36	Parents viewed decision-making as difficult for some adolescents.	Bjonness et. al[7]	U
Illustration 36	One of the tough things for her, and it has to do with her illness, is to make decisions. So, to give her utterly open outcome spaces makes it entirely impossible for her. It is impossible in trivial things, so to give her all the options... She would not be able to answer. But if she could get a kind of		

	delimitation, choose between this and this, then she would probably more easily be able to participate in decisions. (Parent 11)		
Finding 37	Como a outra face da mesma moeda, são os relacionamentos suportivos que amenizam os efeitos das situações adversas vivenciadas, promovendo possibilidades de um crescimento mais saudável ao proporcionar sensações de segurança e confiança.	Rossi et. al[11]	U
Illustration 37	"...para falar sobre o que mais me ajudou ao longo de todo o processo, eu penso muito na minha mãe. Porque quando eu falei pra ela sobre o que estava acontecendo, ela realmente me entendeu e procurou me ajudar. (...) ela não me julga, eu penso muito nisso..."		

Category "Individual therapy"			
		Study	Assessment
Finding 38	In terms of treatment, many parents mentioned that the existing treatment is relatively simple and hoped to increase psychotherapy.	Fu et. al[8]	U
Illustration 38	... At present, it is mainly medication, and I think it still need spiritual communication. At the psychological level, it is necessary to do some intervention and guidance...		
Finding 39	Of these youth, several reported less individual (''one-on-one'') therapy or time with doctors than they needed or expected—they felt that treatment was not sufficiently intense and individualized for them to make progress:	Moses[3]	U
Illustration 39	''I wasn't getting very much one-to-one time which was important to me, and I know it was important to other people there too.'' (Caucasian female, age 16)		
Finding 40	Além disso, a partir do momento em que os adolescentes foram acolhidos pelo CAPSij, identificamos, em seus relatos, a valorização dos espaços de escuta individualizada oferecidos pelos profissionais do serviço, conforme trecho a seguir:	Rossi et. al[11]	U
Illustration 40	"Além da médica, eu venho conversar com uma das referências, com quem eu me vinculei desde a primeira vez, com a outra eu ainda não consegui me abrir, meu vínculo é na verdade com a primeira. Ela até aceitou trocar alguns e-mails comigo, já que eu não queria vir"		
Finding 41	No entanto, nas histórias dos participantes sobre suas experiências no contexto do CAPSij, observa-se a valorização das estratégias individuais em detrimento das outras formas de ofertas terapêuticas de natureza mais coletiva.	Rossi et. al[11]	U
Illustration 41	"Acho que já faz um ano que eu vou no CAPS. Desde que eu entrei lá só passo com a psicóloga e com a médica. Nunca participei de outras atividades"		
Finding 42	Individual therapy	Salamone-Violi et. al[4]	U
Illustration 42	They get me here, so here is where I should get help so that I can cope outside. I know it's not the real world, but it's not as scary, so I think I would feel better, accept more help if it came from here where I truly think people understand.		
Finding 43	Desire for more individual support during inpatient time	Thabrew et. al[6]	U
Illustration 43	'It would have been helpful if I could have had someone to talk to'		

Category "Group therapy"			
		Study	Assessment
Finding 44	Moreover, children are not willing to do physiotherapy and group therapy because these treatments are uncomfortable and innefctive for them.	Fu et. al[8]	C
Illustration 44	... A treatment that shook her ears, she didn't want to do it and said it was uncomfortable...		
Finding 45	Group therapy was explicitly noted by 30% as one of the most helpful aspects of hospitalization because it provided opportunities to ''open up'' to others without shame, listen to others' stories, receive reassurance that hey are not alone, and discuss coping skills	Moses[3]	U
Illustration 45	Group was just good 'cause we all, like, opened up. And a lot of people ended up crying. And we just, like, got through, like, what happened to us. And, like, it just, it was kind of, like, a relief to get it off your chest and, like, inally open up to somebody that, like, as open as we were.		
Finding 46	In the group context, some patients found staff to be too passive or over-analytical, such that groups were 'not safe' places to share information:	Moses[3]	U
Illustration 46	''Basically, you didn't feel like group was a, a particularly safe place to talk about things.''		
Finding 47	Group therapy	Salamone-Violi et. al[4]	U
Illustration 47	When you are feeling really bad, it's good not to talk sometimes. I've done group work before and it never works 'cause that's when I don't want to talk,		

	then none of the other kids want to talk either, so nah, maybe when I start to feel better, but even then, I prefer one on one.		

**Category "Personalized treatment"**			
		Study	Assessment
Finding 48	The parents wanted their adolescents to receive individually tailored treatment, and several had had to fight for their adolescents.	Bjonness et. al[7]	U
Illustration 48	We often experience that they try to use one success story and put it on a second child without asking. Observe, explore what's right for them. It is time-consuming and more expensive, but you get poor results if you do hasty work.		
Finding 49	Many participants reported that they feel powerless, mainly because everyone's disease is heterogeneous and the treatment is not effective for everyone.	Fu et. al[8]	C
Illustration 49	It's when you want to help a patient, but it feels like sometimes you can't help her, there is a feeling of powerlessness...		
Finding 50	In terms of treatment, it is recommended that a complete set of treatment modes specifically for SRBs be developed with individualized treatment methods.	Fu et. al[8]	U
Illustration 50	... I think there is a need for personalized treatment plans because the group therapy we are doing now is similar to health education...		
Finding 51	Also, activities in the hospital were noted by 10% as without therapeutic merit or childish when they did not match perceived developmental level.	Moses[3]	U
Illustration 51	''I felt I was doing preschooler work''		
Finding 52	Similarities and differences in treatment plans	Thabrew et. al[6]	U
Illustration 52	'I was kept in for much longer than the other girls'		
Finding 53	Participants indicated that crisis plans, a required component of community mental health treatment plans, were most often vague.	Walter et. al[10]	U
Illustration 53	Oh, there was always discussion of crisis plans in court, in wraparound. Let me just say: I've never seen one.		
Finding 54	In some cases, however, disruptive child behaviors in groups led to removal or suspension of the child from the group, leaving parents confused about the usefulness of the group.	Walter et. al[10]	U
Illustration 54	They had an incident where he was a bit out of control, and he banged his face on the floor, made his nose bleed. They suspended him for a day ... he is going there to get help with his behavior and when he shows the behavior, they suspend him. What good is that? So I took him out of that.		

**Category "Power of music therapy"**			
		Study	Assessment
Finding 55	Managers reported having their belief in 'music therapy and its potential to improve patient experiences and outcomes' affirmed.	Patterson et. al[12]	U
Illustration 55	Adolescents with mental health issues have trouble expressing emotions and music therapy is valuable for that...it can improve attending...and the social skills component ... helps them to be with other people without making demands.		
Finding 56	Staff, often referencing reluctant engagement of patients in other activities (e.g. cooking groups, gymnasium sessions), described patients as typically enthusiastic about MT, particularly for second and subsequent sessions.	Patterson et. al[12]	U
Illustration 56	They often come up and ask me when the next session is .... It's the only thing they do that for.		
Finding 57	Patients were described as calmer and 'more engaged' after MT.	Patterson et. al[12]	U
Illustration 57	Usually doesn't talk at all but it's like they are different people in that group, some clients hang off every word, it brings the group together and that lasts.		
Finding 58	Music therapy was thought to support expression of difficult emotions in a constructive way.	Patterson et. al[12]	U
Illustration 58	Well, after one session, four or five of them were in their rooms crying, but that was a difficult bunch, really volatile mix, and ...at least they weren't self-harming, much easier to manage crying.		
Finding 59	They described finding themselves singing along with patients, moving to the music, and feeling happy going about their work.	Patterson et. al[12]	U
Illustration 59	We've seen a calm, containing, social component on the unit with music therapy... a positive effect on the patients when it's obvious staff are happy.		
Finding 60	Music Therapy increased participants' comfort in the inpatient setting	Rosado[13]	U
Illustration 60	[Music therapy] just made me feel better and more at home, because I listen to a lot of music at home... you get homesick here.		
Finding 61	Participants processed their experiences outside of the hospital	Rosado[13]	U
Illustration 61	I think that I never realized how much I've actually gone through until I got		

	here. And [in song writing], I just started to think a lot about my actions, and how much I was actually doing to myself.		
Finding 62	Music Therapy encouraged participants to be more engaged in their treatment process	Rosado[13]	U
Illustration 62	Coming out of [music therapy], I felt like the voices in my head were gone. I wanted to feel better and I came out of there with a purpose, knowing that things like that were going to help me in my journey.		
Finding 63	Music Therapy fostered positive peer connections	Rosado[13]	U
Illustration 63	... I just started seeing how other people felt [in music therapy] ... I was like, 'Wow. That hits.' ... I kind of connected with everybody without even talking to them.		
Finding 64	Music Therapy encouraged empathy	Rosado[13]	U
Illustration 64	Just knowing how other people feel sometimes helps you because ... [It's] an emotion that I felt before... sometimes that's a way to help another person.		
Finding 65	Music Therapy modulated affect	Rosado[13]	U
Illustration 65	I felt that [music therapy] really helped my mood go up. Because ... just the satisfaction of hearing it all come together like one big band... we didn't have to try that hard to come all together.		
Finding 66	Music Therapy facilitated catharsis	Rosado[13]	U
Illustration 66	... My personal life, I keep it inside. So [music therapy] brings out a way to like... let it all go. Without even thinking like, 'Oh, I'm just talking about my feelings.' You don't even realize you're doing it.		
Finding 67	Music Therapy helped participants connect to internal resources	Rosado[13]	U
Illustration 67	It was nice to have something to look forward to during the day... so... It kind of made me hopeful. My major problem is that I don't really have enough faith in the future. So to know that I can feel hopeful is pretty... you know... good.		
Finding 68	Music Therapy experiences affirmed music as coping tool	Rosado[13]	U
Illustration 68	[Music therapy] helped me to know that music is probably my best option for when I'm using a coping skill. ...it kind of just lets everything out. But the song is doing it for you.		
Finding 69	Music therapy helped participants discover strenghts	Rosado[13]	U
Illustration 69	It was nice to learn something new... it makes you feel good – it's like 'wow I learned that. That's pretty cool.' ... I think it raises my self-esteem to learn something new.		
Finding 70	Music Therapy helped participants gain insight into their experiences	Rosado[13]	U
Illustration 70	I liked having a song to represent what we thought... Just thinking a lot about my past... [The songs] make me feel closure... it makes me feel like I'm not alone.		
Finding 71	Music Therapy regulated emotions	Rosado[13]	U
Illustration 71	... The music sort of grounded me. Sort of brought me back down to where I was heading... And that really helped a lot. To be able to connect to that.		
Finding 72	Music Therapy increased emotional expression	Rosado[13]	U
Illustration 72	I expressed myself more [in music therapy] than I do when I'm with other people... in music therapy I was able to just kind of follow through with my feelings... I didn't have to cover up my feelings. I was able to kind of just express how I felt.		
Finding 73	Music Therapy affirms participant's strenghts	Rosado[13]	U
Illustration 73	[Connecting with peers] just helps me become a better person. Just knowing that when I feel a certain way, like maybe when I feel depressed or something, or things aren't going right at home, I'm not the only one. Because there are other people here, who are maybe here because of the same reason... And... it kind of built my confidence up. Because I was like, 'well I'm not alone here, there's other people.' So yeah, it just kind of built up.		
Finding 74	Music Therapy affirms coping resources	Rosado[13]	U
Illustration 74	[Music therapy provides a] way to express yourself that's not talking. Because I feel like, when you have a problem, a lot of it is like... talking to another person about how you feel. But sometimes you can't do that. So you can do it in a way that you don't have to say anything.		
Finding 75	Music Therapy integrates cognitive and affective processes	Rosado[13]	U
Illustration 75	Well... I figured out that some of the relationships I was having with people in my life were not healthy. I figured out in some of the songs that I didn't need people in my life that I was holding close. Because they just weren't good for me... And after the lyric analysis, I sort of... especially visually looking at the lyrics... you're able to sort of be like, 'Oh, this person sort of went through the same thing. And they're making a song about it. And it's a real thing. And I need to get rid of that too. Because it's not good for me.'		
Finding 76	Music Therapy provides continuity of experience	Rosado[13]	U

Illustration 76	I came in [to music therapy] kind of nervous and anxious because it was my first day here... I didn't know what was going on. And after that, I felt more relaxed... More content... It was nice to hear the song that I liked and I liked hearing other people's songs.		

**Category "Psychotropic medication"**			
		Study	Assessment
Finding 77	For a few (15%), a most important benefit from hospitalization involved being assessed and prescribed psychotropic medication.	Moses[3]	U
Illustration 77	''I don't think my medication would have gotten figured out right if... if I wouldn't have stayed in there where they could monitor it the entire time.''		
Finding 78	Em relação à medicação, os CAPSij apontam o uso de intervenções medicamentosas quando necessário nas situações de crise.	Moura & Matsukura[9]	U
Illustration 78	[...] Sempre quando ele tem crise a gente traz ele aqui, já passa no médico, ele já dá um diagnóstico, passa remédio para ele e ele acalma. (DSC 4)		
Finding 79	Além da adesão e da frequência efetiva ao serviço, outra dificuldade identificada abarca o uso da medicação, tanto em termos da adesão como de uma perspectiva psicoeducativa e centrada em como os adolescentes percebem o uso da medicação.	Rossi et. al[11]	U
Illustration 79	"...Eu continuo indo [ao CAPSij], mas eu acho que eu não preciso de remédio. Eu tomo! Mas não preciso mais (...). Se eu tivesse que falar alguma coisa para as profissionais do CAPS, falaria pra não passarem tanto remédio. Eu acho que eu tomo muito remédio, dá muito sono em mim, e é ruim sentir muito sono (...). Fica difícil fazer as coisas, não dá vontade de ir pra escola..."		

**Category "Family intervention"**			
		Study	Assessment
Finding 80	The parents emphasized a need for family interventions.	Bjonness et. al[7]	U
Illustration 80	It has been a long process to understand what the disease entails. I feel that being part of a family group helped to get some of those answers. Together with other families in the same or similar situations, we could share experiences.		
Finding 81	Family meetings were reported by 6% to be unhelpful in the following situations: (a) when there was arguing/fighting with no attempt at resolution; (b) when either parents or staff (or both) did not listen to the youth; and (c) when staff sided with parents:	Moses[3]	U
Illustration 81	''...The family meeting made me super upset... And I don't, I don't like her (therapist) 'cause she's not, like, trying to listen to my side of the story or anything.''		
Finding 82	Family meetings	Salamone-Violi et. al[4]	U
Illustration 82	My parents are divorced and they hate each other. They don't even want to be in the same room together, so here on the ward they have to, they have to listen and talk.		

**Category "Restraint"**			
		Study	Assessment
Finding 83	The physical and interpersonal and physical hospital environment was notably helpful to 34% of the participants.	Moses[3]	U
Illustration 83	''... no matter I wanted to (cut) so bad, I couldn't 'cause there was nothing there. Because of that I end up not cutting, no matter how bad I wanted to.''		
Finding 84	A similar number reported feeling frightened when witnessing other children either (a) restrained by staff	Moses[3]	U
Illustration 84	''I saw them tie a little girl in restraints... that would be weird to anyone'' (Caucasian male, age 15);		
Finding 85	A contenção é citada pelos familiares como uma estratégia de cuidado realizada pelos CAPSij em situações limite e que também pode gerar sofrimento nos familiares, conforme apresenta o DSC 5.	Moura & Matsukura[9]	U
Illustration 85	Uma vez ele quebrou um vidro com as mãos e ficou muito nervoso, eles levaram ele pra uma sala e seguraram, a técnica falou: - Pode ir embora ele vai ficar bem, nós vamos conversar com ele! - Eu fui embora, mas do outro lado da rua eu escutava os gritos dele. Naquele dia eu fui embora muito deprimida. Aí, eu liguei e ela falou: - Ele já está bem! Está brincando, já comeu! Não está mais em crise não. (DSC 5)		
Finding 86	Containment	Salamone-Violi et. al[4]	U
Illustration 86	At home... it exhausts me. I have to keep me and other people safe. Here I don't have to worry about myself.		

**Category "Community mental health service treatment"**

		Study	Assessment
Finding 87	De acordo com quadro 1, outra estratégia bastante citada pelas equipes é o acolhimento diurno (hospitalidade diurna), representado pela ambiência/convivência, um dispositivo fundamental na atenção à crise e pouco discutido no campo da saúde mental infantojuvenil.	Moura & Matsukura[9]	U
Illustration 87	Principalmente quando ele teve crises. Eles me ajudaram muito. Eu vinha três vezes por semana, toda semana e ele ficava aqui, elas ficavam com ele e davam a maior atenção.		
Finding 88	O CAPS III atende prioritariamente pessoas em intenso sofrimento psíquico e proporciona atenção contínua, com funcionamento 24 horas, ofertando retaguarda clínica e acolhimento noturno a outros serviços de saúde mental do território (BRASIL, 2002; 2011).	Moura & Matsukura[9]	U
Illustration 88	Trata-se de um poderoso recurso para lidar com situações de crise, prevenindo internações hospitalares, possibilitando intervenções mais humanizadas, uma vez que o acolhimento à demanda de sofrimento apresentada pelo sujeito pode ocorrer fora do período comum aos CAPSij II que são das 07:00 às 19:00 de segunda a sexta-feira. (Gestor A)		
Finding 89	O DSC 7 levanta a necessidade de que alguns recursos internos e externos estejam disponíveis às equipes de CAPSij, a fim de garantir uma melhor estrutura para que esse equipamento acolha de forma resolutiva as situações de crise de crianças e adolescentes de seu território.	Moura & Matsukura[9]	U
Illustration 89	Assim, quando a criança tivesse em crise que eles [CAPSij] tivessem um acolhimento melhor. [...] Aí, você fica perdida porque você não sabe onde leva a criança e outra, você não acha socorro de lado nenhum. Igual o meu neto, ele teve uma crise muito forte, a moça ficou correndo com ele em vários hospitais e ninguém aceitava, entendeu? Aí foi umas três horas da manhã um único hospital que ficou com dó ainda, segurou ele lá, mas depois mandaram ele vir para o CAPS porque o CAPS é que tinha que dar assistência para ele, mas eles não têm como dar assistência. Porque se uma criança ficar ruim aqui, eles não têm como, não tem ambulância, não tem nada, não tem um médico de plantão 24 horas para atender. [...] Também, se você vai para o hospital a maioria que tem aí é tudo adulto. Eles não atendem crianças. Aí fica difícil. Ele ficou indo em vários hospitais e tudo longe[...] e a criança vai ficando mais agitada ainda. [...] É terrível. Eles jogam tudo misturado. Eu acho que o CAPS tinha que ter um lugar próprio para receber essas crianças.		
Finding 90	A partir dos resultados aqui apresentados, verifica-se que as equipes refletem sobre a diferença de cuidado proporcionada por um acolhimento noturno e por uma internação hospitalar.	Moura & Matsukura[9]	U
Illustration 90	O CAPSij III apresenta-se como mais um valioso recurso para o enfrentamento do sofrimento psíquico e na luta pela garantia dos direitos de crianças e adolescentes. Um ponto muito importante na compreensão deste dispositivo é a distinção entre acolhimento noturno e o processo de internação. Na internação o sujeito é apartado do convívio social com o objetivo da atenuação dos sintomas. Já no acolhimento noturno, os pressupostos da clínica comunitária levam em consideração a permanência do sujeito o mais próximo possível de seu território e das pessoas de sua convivência, mantendo a autonomia do sujeito, respeitando seus desejos e fragilidades e construindo seu projeto terapêutico singular com corresponsabilidade.		

**Category "Lack of privacy"**			
		Study	Assessment
Finding 91	However, for some participants, the "24/7" support felt too much, particularly at times when they wanted to be on their own	Gill et. al[1]	U
Illustration 91	No ones has time to spend alone, because as you can see all these doors are locked. You can't go into your bedroom... the only place you could possibly have all by yourself is the loo.		
Finding 92	Staff behavior perceived as unhelpful included: intrusion on space (e.g., ‘‘she got right up in my face, not even half a foot away from me’’ (001)); meanness, disrespect or impatience:	Moses[3]	U
Illustration 92	‘‘a few of them would, like, they would, like, yell at certain kids, like, like, other teens and stuff, and, you know, like, for something that wasn't necessary. Like, they didn't have the patience...’’ (Caucasian male, age 16),		
Finding 93	Environment	Salamone-Violi et. al[4]	U
Illustration 93	The whole time I was here for awhile I was thinking that, I don't know what the word is, confined maybe in a sense, because you know sometimes they lock the doors because some people are here against their will, and I felt confined.		
Finding 94	Lack of privacy in hospital	Thabrew et. al[6]	U

Illustration 94	'She (my neighbor) would be hearing all of my conversations and I could hear all of her conversations'		
Finding 95	Restriction of personal freedom	Thabrew et. al[6]	U
Illustration 95	'I wasn't allowed to do anything'		

Category "Stigma"			
		Study	Assessment
Finding 96	A few participants mentioned that the lack of social recognition and acceptance of psychiatric patients is a major challenge. It is difficult for these patients to return to society after discharged.	Fu et. al[8]	U
Illustration 96	" ... The whole society still has a certain prejudice against mental illness. They cannot get social approval and support ... " (Psychiatrist, p3, p4)		
Finding 97	Prior to their admission, several identified with the stereotypes of "madness", expecting lots of "crazy people to run around screaming".	Gill et. al[1]	U
Illustration 97	Crazy people to run around screaming		
Finding 98	Through their experiences they had learnt that people with mental health problems are "like everybody else", which had not only impacted on their overall view of mental health, but also helped them to re-evaluate their beliefs about their own problems.	Gill et. al[1]	U
Illustration 98	You come here and realise [a mental health problem] is not a bad thing, it can happen to anybody. And that makes it feel less harsh on yourself... it doesn't make me a freak, it doesn't make me any less of a person than anyone else who doesn't have a mental health disorder.		
Finding 99	Being admitted to a mental health unit in particular appeared to contribute to the deterioration of friendships, with participants experiencing misunderstanding, stigma and prejudice from peers	Haynes et. al[2]	U
Illustration 99	I don't think many people are quite open-minded about people going to a Psychiatric hospital. I think they probably thought I was going crazy or something... I think maybe they just wanted to distance themselves from me bacause of it.		
Finding 100	Only three youth responded to the research questions by reporting fear or concerns about stigma.	Moses[3]	U
Illustration 100	‘‘Like, 'cause it's called a mental hospital and that kinda makes you feel like if you're there, you must be mental, you must be crazy."		
Finding 101	Fear of the unknown	Salamone-Violi et. al[4]	U
Illustration 101	I didn't know what to expect. I didn't know if people were going to like me being there and like me as a person.		

Category "Exclusion of everyday life"			
		Study	Assessment
Finding 102	The young people described the inpatient environment as strikingly different from their home life or "the outside world".	Gill et. al[1]	U
Illustration 102	A fake world		
Finding 103	The sense of living in an alternative reality permeated all participants' accounts of their experience of hospitalisation.	Haynes et. al[2]	U
Illustration 103	It was just like completely different to anything else I've ever experienced, it's like I don't know, I just can't put it into words, how different it was.		
Finding 104	Hospitalisation was therefore experienced as an interruption to daily life, which caused participants to miss out on particular events or valued aspects of their lives.	Haynes et. al[2]	U
Illustration 104	I should've been to college and I should be doing all these things that other people my age are doing, and I feel a bit trapped in here in that.		
Finding 105	Participants spoke about the importance of taking part in normal everyday activities on the ward, which was understood as an attempt to recreate a familiar reality within the unfamiliar context of the ward environment.	Haynes et. al[2]	U
Illustration 105	If I was at home I would be the man of the family, and I look at the lads on the unit as my family, so you know, when one of them was getting attacked it was basically as if your're saying you know, my mother or my brother as etting attacked. So Id just forget you know, that they were patients, and split em up.		
Finding 106	The opportunity to experience a ‘‘time-out'' was critical for almost a third (29%) who reported that reflecting on their problems and circumstances without everyday pressures was one of the most helpful aspects of brief hospitalization:	Moses[3]	U
Illustration 106	‘‘(It was) good to step out of life for a little bit and then get better'' (Caucasian female, age 14);		
Finding 107	Ward activities	Salamone-Violi	U

Illustration 107	It's really boring. If I was here on my own, it would be a hell hole. There's not much to do.	et. al[4]	
Finding 108	Limitations to freedom	Stanton et. al[5]	U
Illustration 108	It's frustrating being locked up, being restricted to a lot of things . . . missing out on life, not experiencing what a normal teenager should experience.		
Finding 109	Removal from normal life	Thabrew et. al[6]	U
Illustration 109	'I was in hospital, away from my friends, away from my regular life and doing something that caused a lot of mental stress' (P3)		

**Category "Hospital routine"**			
		Study	Assessment
Finding 110	The high level of structure and routine on the unit (e.g. fixed meal times and bedtime) was a novel experience for most participants, who were used to doing "what I wanted when I wanted".	Gill et. al[1]	U
Illustration 110	Routine's important, it is necessary for managing yourself... the devil makes work for idle minds, but here you're always two minutes away from something else to keep you busy		
Finding 111	Participants described feeling restricted in a number of ways during admission, which could be viewed on a continuum.	Haynes et. al[2]	U
Illustration 111	I got quite violent, um towards the staff because I saw them as keeping me prisoner, keeping me prisoner inside this place... I just felt really hostile towards the staff for keeping me here, for locking me in.		
Finding 112	Several indicated that the simplicity of being in an environment where their time was completely structured served to create inner peace and distract from their troubles:	Moses[3]	U
Illustration 112	The nurses there always came and checked up on you or someone every fifteen minutes, every half an hour came in and said, ''Time for this class or time for that class.'' ...they would come in and check on you and be like, ''Just wondering where you were'', or ''Wondering what you're doing.''... (I: Was that good or bad?). Um, I felt that kind of actually, like, kept me safe. I thought that was good. (Asian female, age 16)		
Finding 113	More generally, complaints for 12.5% revolved around the ''strict'' and regimented schedule they were ''forced'' to participate in.	Moses[3]	U
Illustration 113	Like, even in school you're not, like, time! Time! Time! Time! Like, you can be late. But if you were late (in the hospital), they'd be like, ''Hah, hah, hah. Where were you? You get 0 points for the day!'' (Caucasian female, age 15)		
Finding 114	Restrictions on verbal interactions and restricted physical contact with peers were noted as particularly difficult and perplexing as such interactions were perceived by some as a potential source of comfort and ''healing'':]	Moses[3]	U
Illustration 114	I just thought that it, like, all the rules were kind of ridiculous and stuff. Like, no touching and stuff, like, I don't know. I feel like it's those times when you need, like, a hug the most, you know?		

**Category "Distance from beloved ones"**			
		Study	Assessment
Finding 115	For many participants feeling disconnected from family was the most difficult aspect to being in hospital and was associated with feeling isolated and alone.	Haynes et. al[2]	U
Illustration 115	Every single day my sister's asking for me to come and see her, but she just doesn't realise I can't come see her... I was the man of the family, the very time the family needed me I was locked up.		
Finding 116	Participants claimed that these periods of 'alone time', when not allowed to interact with peers were often experienced as lonely and even frightening, especially when rooms were perceived as plain and stark with few personal belongings:	Moses[3]	U
Illustration 116	''Being alone sometimes was really bad. You'd feel, like, so alone and with your own feelings and you'd just get so depressed.'' (Caucasian female, age 14)		
Finding 117	Several participants noted being distressed or anxious about being away from home:	Moses[3]	U
Illustration 117	''It kinda made me feel down 'cause I mean, you get this room and it's not your room and you don't get to see your family or your animals.'' (mixed race female, age 17)		

**Category "Fear of returning to inpatient unit"**			
		Study	Assessment
Finding 118	The prospect of returning to the inpatient unit symbolised a personal failure	Gill et. al[1]	U
Illustration	"back to square one" (P1, P5, P7).		

118			

Category "Coping skills"			
		Study	Assessment
Finding 119	Participants felt they had developed a better understanding of their difficulties, which had helped them build a more positive self-concept.	Gill et. al[1]	U
Illustration 119	All these little things all kind of add up to one big change... I feel like I've been transformed in a way, like upgraded to a new me.		
Finding 120	Several participants described having learnt strategies to manage their difficulties, with one referring to her newly acquired coping strategies as "survival skills".	Gill et. al[1]	U
Illustration 120	Survival skills.		
Finding 121	Participants employed a number of intellectual strategies to survive their time in hospital, including normalising, rationalising, looking forward and thinking positively.	Haynes et. al[2]	C
Illustration 121	I just think well, you know, that was then, and if you know they were like that, then they couldn't have been real friends.		
Finding 122	Finally, avoidance was an important coping strategy for some participants, particularly in terms of coping with the violence and self-harm they witnessed as an inpatient.	Haynes et. al[2]	U
Illustration 122	It's hard when youre living with all that violence. I would just try and shut off. I'd disappear into my own little world, my own bubble and just exist in that bubble.		
Finding 123	Over half (56%) spoke of gaining cognitive and behavioral coping skills which allow them to better anticipate their reactions to things and people as well as utilize a new repertoire of strategies to ''to pick out the important things to focus on'' (African American female, age 16) and prevent escalation or self-harm	Moses[3]	U
Illustration 123	Well, the coping skills I learned that if I do get overwhelmed what can I do to help me challenge that. Like what can help me solve the problem without cutting myself, shooting myself, or hurting myself in any physical way, or hinking about suicidal, or thinking about hurting somebody else. That was really helpful because I didn't know how to do any of that. And what I learned was I can go for walks, I can listen to music, I can read, I can – you now, I have a friend I can ask for help if I ever need it.		
Finding 124	Nas histórias narradas foi possível observar a descrição clara de sensações de desespero, confusão mental, angústia, sentimento de inferioridade, baixa autoestima, medo e descontrole, bem como de comportamentos impulsivos, disparados por tais sensações desconfortáveis, atrelados, muitas vezes, a pensamentos relacionados à morte.	Rossi et. al[11]	U
Illustration 124	"Qualquer coisa que acontecia eu achava que eu era um inútil, um imbecil, achava que as coisas nunca iam dar certo, me inferiorizava em todos os aspectos. Parava pra pensar no dia a dia, na vida, no mundo lá fora, e concluía que isso aqui não vale nada também. (...) Um belo dia eu saí pra trabalhar, normalmente, acordei como em um dia normal, com a cabeça cheia de coisas, e simplesmente me deu um estalo assim, eu pensei, por que eu não morro logo? E aí eu fui pra avenida mais movimentada da cidade, em que os carros passam em alta velocidade. Fiquei parado no meio dela e os carros começaram a desviar de mim"		
Finding 125	Assim, relacionamentos familiares e interpessoais quando vivenciados como relacionamentos violentos, nas diferentes formas de violência, e inclusive aqueles que repercutem no sujeito como desvalor e abandono objetivo e subjetivo/emocional, foram identificados como fatores de desencadeamento e até de causa do sofrimento psíquico.	Rossi et. al[11]	U
Illustration 125	"...meu pai passou a não gostar mais de mim e das minhas irmãs. Daí eu vivo brigando com ele, sempre pelo mesmo motivo, por achar que ele não gosta de mim, mas esse dia eu estava com muita raiva. Isso foi em dezembro do ano passado, eu acho. Nós brigamos e eu tomei inseticida"		

Category "Discharge planning"			
		Study	Assessment
Finding 126	Young people identified the need for others to support them when they were discharged from the inpatient unit, including their family, friends, and community mental health teams to get out of. (P10)	Gill et. al[1]	U
Illustration 126	"knowing that I've got a good support network out there waiting for me" (P4).		
Finding 127	All the young people desperately wanted to be seen as "normal" by their	Gill et. al[1]	U

	friends and others in their community when they were discharged from the unit.		
Illustration 127	People might get worried in the sense that I'm not the same person anymore, that I'm different. But I don't want to be perceived as that. I just want to be normal. (P1)		
Finding 128	Further, looking forward and thinking positively appeared to be particularly useful in helping participants to think about the future and to minimise the overall impact of hospitalisation.	Haynes et. al[2]	U
Illustration 128	I: How have you kind of managed those feelings about having missed out on college and things? P: I just reassure myself I can always do it another time. Try and think positive and look forward.		
Finding 129	Discharge planning	Salamone-Violi et. al[4]	U
Illustration 129	Maybe during that time I would try having some leave, see if it's OK, then have more leave, go see some friends, do what I do normally, maybe stay overnight and then go after that.		
Finding 130	Transition home	Thabrew et. al[6]	U
Illustration 130	'I quite liked having meals off the ward. It was less pressure because there were less people around'		

Category "Acceptance"			
		Study	Assessment
Finding 131	Unlike patients with physical illness, some patients do not feel sick and therefore think they do not need treatment.	Fu et. al[8]	U
Illustration 131	"The patient doesn't understand and doesn't think he is sick, so his desire for treatment is not high, and then the doctor's sense of accomplishment is relatively low." (Psychiatrist, p5)		
Finding 132	Os adolescentes abordaram a crise e seu entorno de forma individualizada, tratando-a como uma questão essencialmente interna, como algo que criaram e que, portanto, devem superar sozinhos.	Rossi et. al[11]	U
Illustration 132	"O fato de eu estar bem ou mal está ligado somente a mim. É claro que tem coisas que acontecem no dia a dia que deixam a gente mal ou bem, mas o fato de eu estar bem ou mal da cabeça, se atribui única e exclusivamente a mim"		
Finding 133	Acceptance	Salamone-Violi et. al[4]	U
Illustration 133	I dunno if I was hoping it would happen, I just, well I just wanted to feel better and I needed help, so yeah, I guess it was alright.		
Finding 134	Powerlessness	Salamone-Violi et. al[4]	U
Illustration 134	I was in my bed. I didn't want to, I didn't want them there, but they picked me up and carried me to the ambulance.		
Finding 135	Relatedness	Stanton et. al[5]	U
Illustration 135	I don't have to hide anything in here, we just be who we are without worrying about what people think of us.		
Finding 136	Being re-fed	Thabrew et. al[6]	U
Illustration 136	'Getting the NG tube was really scary; it made it feel more real'		
Finding 137	Being treated against my will	Thabrew et. al	U
Illustration 137	'I kind of felt I was being punished the whole time'		

Category "Adolescents' decision-making"			
		Study	Assessment
Finding 138	The parents referred to self-determined treatment as adolescents' ability to make decisions and take responsibility.	Bjonness et. al[7]	U
Illustration 138	I experience that she agrees and participates in decisions herself (...) Crucial for success is that they agree and are ready to contribute to the treatment		
Finding 139	The parental dilemma was the extent to which they should strengthen their adolescents' autonomy by allowing them to make decisions.	Bjonness et. al[7]	U
Illustration 139	It's like a double-edged sword. I have experienced that she often says no, but she is actually happy about it. It just takes a while before she sees it for herself. (Parent 9)		
Finding 140	Although the parents claimed to know what was best for their children, they admitted that the treatment had poor utility without the adolescents having a say in decision-making.	Bjonness et. al[7]	U
Illustration 140	We have learned to keep our hands behind our back... The most challenging during admission was to let him participate in the treatment without influencing him about what is best. We must leave the choices to him. It's his role, not mine, although I fear and dread of where it might lead him. It's terribly difficult not to be part of decisions, even though you know you must		

	leave it to him.		
Finding 141	Other treatment decisions related to treatment plans (e.g., restriction on talking with certain individuals, recommendation to eliminate computers from the house) or to the focus of intervention:	Moses[3]	C
Illustration 141	‘‘...they wanted to create safety plans and stuff like that so I wouldn’t hurt myself, or anything. But, like, I don’t know, I felt that they were kinda pointless.’’ (Caucasian female, age 17)		
Finding 142	For others (5.0%), what frightened them was not knowing when they would be released, along with the possibility that they would be in the hospital for a ‘‘long time’’:	Moses[3]	U
Illustration 142	‘‘I just thought I was going to be there for a long time...weeks, I thought I was gonna be there for weeks.’’		
Finding 143	Having a voice and choice	Stanton et. al[5]	U
Illustration 143	It feels like I’m just talking to someone that’s just taking information and receiving it. They’re not really giving me any feedback		
Finding 144	Not feeling heard	Thabrew et. al[6]	U
Illustration 144	‘It really frustrated and upset me because they would talk to my parents, but they never talked to me’		
Finding 145	Not feeling included in management decisions	Thabrew et. al[6]	U
Illustration 145	‘I would definitely have appreciated being included more. Maybe asked; I mean, I don’t really know how to fix the problem, I just know that I didn’t appreciate being told what to do and not being included.’		

**Category “Treatment outcomes”**			
		Study	Assessment
Finding 146	Furthermore, a few participants believed that their children's behavior is not a natural manifestation but a response after treatment.	Fu et. al[8]	U
Illustration 146	... I feel like she's a little more excited now. Because she wouldn't do that kind of intimate behavior to me before, but she would do it deliberately now...		
Finding 147	Although the majority of participants described significant improvement in their emotional wellbeing, all believed they would be leaving the unit with some ongoing difficulties.	Gill et. al[1]	U
Illustration 147	I'll struggle in some areas [of emotional wellbeing] but I think they'll be a lot easier to get out of.		
Finding 148	In light of anticipating continued difficulties after discharge, a common aim in thinking about leaving was to take to things slowly, "one step at a time".	Gill et. al[1]	U
Illustration 148	Before I was the sort of person that would jump straight into something... Now I'm thinking maybe I should take baby steps... it would be much easier than taking a big long jump and then breaking down again.		
Finding 149	Others reported feeling anger or disappointment when discharged before they perceived themselves as ready:	Moses[3]	U
Illustration 149	So I truly was not even ready, and I was not even expecting to get discharged...(I) ended up getting discharged sooner and... I thought, like, I wanted to be ready. Like, for my parents I wanted to be ready, but I’m not.		
Finding 150	Mental state	Salamone-Violi et. al[4]	U
Illustration 150	My calming down, I'm more calm, not going off at people, and talking to my psych who comes in to see me on the ward has been good		
Finding 151	Competence	Stanton et. al[5]	U
Illustration 151	I feel significantly more confident than I did before coming in here. I still feel emotional, sad and anxious but I feel like I’ve learned a lot of things and lessons here that over time I will be applying that will help me as an individual just cool off and be a generally healthier person.		

**Category “Sense of security”**			
		Study	Assessment
Finding 152	Feelings of safety and security on the inpatient unit were common amongst the young people, with one describing the experience as if she were "wrapped in cotton wool".	Gill et. al[1]	U
Illustration 152	We're being watched quite a lot of the time... I think it's quite good because I don't have a chance to hurt myself and I know I'm safe.		
Finding 153	Some talked about the safety of the unit as a double-edged sword: it provided a high level of security whilst on the unit, yet gave a "false sense of security" about the "real world", where they would be exposed to difficulties that they would not be prepared for.	Gill et. al[1]	U
Illustration 153	False sense of security.		
Finding 154	A relatively small number of participants also related feeling anxious or	Moses[3]	U

	scared in relation to circumstances or activities in the hospital.		
Illustration 154	‘‘They just kept asking questions, and I didn’t like it’’		
Finding 155	A safe place to stay	Thabrew et. al[6]	U
Illustration 155	‘It kind of became your safe haven’		

**Category “Improvement of labor process”**			
		Study	Assessment
Finding 156	They suggested increasing staffing and optimizing the division of labor.	Fu et. al[8]	U
Illustration 156	... We do not have much time to communicate with patients and solve some of their psychological problems. Understaffing is a factor, and the second may be the division of labor is not optimized...		
Finding 157	Some participants believed that supervision and training on SRBs are also needed.	Fu et. al[8]	U
Illustration 157	... I think we need a professional psychotherapist to supervise us. Now we are just exploring some communication skills and psychological intervention methods ourselves.		

**Category “Relationship between family members and health services”**			
		Study	Assessment
Finding 158	Several parents pointed out that they had to coordinate their son's or daughter's health services themselves.	Bjonness et. al[7]	U
Illustration 158	We had to be the mediator between them (different healthcare services) on things they ought to know. It is silly because it takes a lot of energy, and it has taken a long time for us to understand the system. Who is really responsible, and who should take the initiative? In the end, we have to do it.		
Finding 159	Compreende-se que os resultados revelam que as equipes dos CAPSij têm pautado suas ações nas diretrizes da atenção psicossocial que prevê o acolhimento imediato ao usuário e sua família, a intensificação do cuidado, trabalho em equipe e a articulação em rede (BRASIL, 2004; 2005).	Moura & Matsukura[9]	U
Illustration 159	Ah! Quando ele está meio assim, ou se acontecer algo em casa e ele ficar muito alterado, eu posso trazer ele aqui. [...] Quando deu a crise nele, ele ficou vindo a semana toda. [...] E ajudou porque ele voltou rapidinho. O médico disse que era de 30 a 45 dias para a mente dele voltar e ele voltou com 15 dias, então voltou rapidinho.		
Finding 160	Fernandes & Matsukura, ao buscarem conhecer os contextos de inserção e as relações sociais estabelecidas por adolescentes frequentadores de um CAPSij, identificaram que os profissionais do serviço ocupavam um l lugar de destaque na rede de suporte relacional/social dos adolescentes e o serviço foi apreciado como o principal contexto de inserção. Esse lugar de destaque também é dado pelos adolescentes deste estudo.	Rossi et. al[11]	U
Illustration 160	“...vim pra cá pro CAPS, melhorei aqui. (...) porque aqui eles me ajudaram pra caramba”		
Finding 161	Common barriers affecting service quality in mental health, child welfare, and school systems alike included a lack of available or accessible services at critical points in children's lives, the lack of communication and collaboration within and between service systems, families' difficulties with transportation and financial resources, and a sense of not being taken seriously.	Walter et. al[10]	U
Illustration 161	I was trying to get him into the mental health center ... he had an appointment. And then a few days before his appointment, they told me that person had been let go. They would have to reschedule another appointment. Well, you can't just reschedule in a couple of days. They make it another month. And by this time, he was out of control again.		

**Category “Emergency as mental health service”**			
		Study	Assessment
Finding 162	Ainda que a atenção à crise deva se dar prioritariamente nos CAPSij, o DSC 6, a seguir, revela que os familiares buscam os Hospitais Gerais nas situações de crise, o que reforça a necessidade de que sejam concretizadas estratégias que favoreçam a implementação da atenção psicossocial no cuidado à crise em todos os equipamentos da RAPS.	Moura & Matsukura[9]	U
Illustration 162	Porque sempre quando dá essas crises, eu já estou longe, quase nunca dá aqui [CAPSij]. Quando vem dá, é na minha casa ou no meio da rua. Sempre é mais a noite e tá fechado também. A gente vai conseguir em casa mesmo ou já procuro recurso em outros hospitais. Aí os médicos já conversam e comunicam aqui, eles respondem pra mim: - Aqui não é serviço da gente é serviço do CAPS. - Aí eu passo pro CAPS o que aconteceu com ele.		

Finding 163	Embora os familiares busquem os serviços que compõem os pontos de atenção de urgência e emergência, nem sempre recebem o cuidado esperado, exemplificado pelo SAMU, que não atende ao chamado, e pelo Pronto-Socorro, que apenas administra a medicação e na sequência libera o usuário para casa.	Moura & Matsukura[9]	U
Illustration 163	Ele tinha 4 para 5 anos, ele acordou e disse que tinha um monte de bicho no corpo, ele rolava no chão, arrancou a roupa todinha e a gente não podia tocar nele que ele gritava. E dava ânsia e ele falava que estava cheio de bicho na garganta dele, ele entrou em pânico. A primeira coisa que eu me lembrei foi de enrolar ele num lençol e levar para o hospital. O médico disse que ele estava tendo uma crise de esquizofrenia, eles deram uma injeção de calmante que não fez efeito e ele ficou a noite inteira gritando. Aí deram mais um medicamento foi aí que ele ficou grogue e ele conseguiu dormir. Eu trouxe ele embora pra casa dormindo. No outro dia foi tudo a mesma coisa.		
Finding 164	Using the ER as an entry point tended to lengthen the process further due to added waiting periods caused by additional paperwork, priority of incoming emergency cases, and procedures, such as required doctor-to-doctor calls before transferring a child out of a medical facility.	Walter et. al[10]	U
Illustration 164	We were [at the hospital ER] for like about an hour and a half before [the screener] even showed up because nobody had informed him that we were there yet. Since we had to go through the emergency room, there was a long wait in the emergency room. We went up there at, it was between 8:00 and 9:00 a.m. And we didn't get out of there until about 2:00 or 3:00 in the afternoon.		
Finding 165	For children in state custody, the admission process was further complicated when procedures and policies to coordinate admissions between the mental health and child welfare systems were unclear to the persons involved, or child welfare agency workers were not readily available to arrange for court orders and signatures on hospital admission paperwork.	Walter et. al[10]	U
Illustration 165	I filled out 5 hours worth of papers. And I did all that. And then it came down to "wait, you're not his guardian, this is all null and void." So they had to send the papers or fax them, to the [child welfare agency] to get them signed. ... So I spent all that time, and it frustrated me, I was thinking: [the child welfare agency] knew that. Why weren't they there?		

**Category "Assessment of the staff"**			
		Study	Assessment
Finding 166	Other participants mentioned that their sense of achievement feels exceptionally low.	Fu et. al[8]	U
Illustration 166	... I sometimes go to speak with patients, but there is no obvious effect for many times, and the patient's conditions is not good. Then he couldn't understand what you were saying and couldn't follow your instructions, so I felt frustrated...		
Finding 167	Most participants mentioned that medical staff with patience, love, a calm personality, and a certain degree of discrimination and empathy are more qualified for working with SRBs adolescents.	Fu et. al[8]	U
Illustration 167	... should be stronger inside and have the ability to distinguish, otherwise you will be led by the patient. Love and empathy are also required.		
Finding 168	Many participants said that working with teenagers who display SRBs requires strong communication and professional skills, and mastering these skills is a challenge.	Fu et. al[8]	U
Illustration 168	... We have to learn communication skills, and the learn some psychological counseling methods to empathize with patients, which is very difficult to learn...		
Finding 169	Perceived deficiencies in staff skills and attitudes	Thabrew et. al[6]	U
Illustration 169	'I kind of felt the (paediatric) nurses weren't trained in mental health'		

*Assessment of studies' credibility according to JBI Critical Appraisal Checklist for Qualitative Research[14].

U: unequivocal; C: credible.

References:

1. Gill F, Butler S, Pistrang N. The experience of adolescent inpatient care and the anticipated transition to the community: Young people's perspectives. J Adolesc. 2016;46.

2. Haynes C, Eivors A, Crossley J. “Living in an alternative reality”: Adolescents’ experiences of psychiatric inpatient care. Child Adolesc Ment Health. 2011;16(3).
3. Moses T. Adolescents’ perspectives about brief psychiatric hospitalization: What is helpful and what is not? Psychiatr Q. 2011;82(2).
4. Salamone-Violi GML, Chur-Hansen A, Winefield HR. “I don’t want to be here but I feel safe”: Referral and admission to a child and adolescent psychiatric inpatient unit: The young person’s perspective. Int J Ment Health Nurs. 2015;24(6).
5. Stanton J, Thomas DR, Jarbin M, MacKay P. Self-determination theory in acute child and adolescent mental health inpatient care. A qualitative exploratory study. PLoS One. 2020;15(10 October 2020).
6. Thabrew H, Mairs R, Taylor-Davies G. Young people’s experiences of brief inpatient treatment for anorexia nervosa. J Paediatr Child Health. 2020;56(1).
7. Bjønness S, Grønnestad T, Johannessen JO, Storm M. Parents’ perspectives on user participation and shared decision-making in adolescents’ inpatient mental healthcare. Heal Expect. 2022;25(3).
8. Fu X, Yang J, Liao X, Shen Y, Ou J, Li Y, et al. Parents’ and medical staff’s experience of adolescents with suicide-related behaviors admitted to a general hospital in China: qualitative study. BMC Psychiatry. 2021;21(1).
9. Moura BR, Matsukura TS. Atenção à crise de crianças Attention to the crisis of children and adolescents: care strategies of Psychosocial Care Centers for Children and Adolescents from the perspective of managers and family members. Physis Rev Saúde Coletiva. 2022;32(1):2–27.
10. Walter UM, Petr CG, Davis S. Informing best practices for children in psychiatric crises: Perspectives and insights from families. In: Families in Society. 2006.
11. Rossi LM, Marcolino TQ, Speranza M, Cid MFB. Crisis and mental health in adolescence: the story from the perspective of those who live it. Cad Saude Publica. 2019;35(3).
12. Patterson S, Duhig M, Darbyshire C, Counsel R, Higgins NS, Williams I. Implementing music therapy on an adolescent inpatient unit: A mixed-methods evaluation of acceptability, experience of participation and perceived impact. Australas Psychiatry. 2015;23(5).
13. Rosado A. Adolescents’ experiences of music therapy in an inpatient crisis stabilization unit. Music Ther Perspect. 2019;37(2).
14. Lockwood C, Porritt K, Munn Z, Rittenmeyer L, Salmond S, Bjerrum M, et al. Chapter 2: Systematic reviews of qualitative evidence. In: Aromataris E, Munn Z, editors. JBI Manual for Evidence Synthesis [Internet]. 2020. Available from: https://doi.org/10.46658/JBIMES-20-03
